# Traditional Applications, Phytochemical Constituents, and Pharmacological Properties of *Lavandula multifida* L.: A Review

**DOI:** 10.3390/molecules30193906

**Published:** 2025-09-28

**Authors:** Mohammed Allouani, Noui Hendel, Dahou Moutassem, Madani Sarri, Djamel Sarri, Antonella D’Anneo, Giuseppe Gallo, Antonio Palumbo Piccionello

**Affiliations:** 1 Microbiology and Biochemistery Departement, Faculty of Sciences, University Pole, Road Bordj Bou Arreridj, M’sila 28000, Algeria; 2 Laboratory of Biology: Applications in Health and Environment, University Pole, Road Bordj Bou Arreridj, M’sila 28000, Algeria; 3 Laboratory of Characterization and Valorization of Natural Resources, Faculty of Nature and Life Sciences, Mohamed El Bachir El Ibrahimi University, Bordj Bou Arreridj 34000, Algeria; dahou.moutassem@univ-bba.dz; 4 Department of Nature and Life Sciences, Mohamed Boudiaf University, M’Sila 28000, Algeria; madani.sarri@univ-msila.dz (M.S.); djamel.sarri@univ-msila.dz (D.S.); 5 Laboratory of Biochemistry, Department of Biological, Chemical and Pharmaceutical Sciences and Technologies (STEBICEF), University of Palermo, Via del Vespro 129, 90127 Palermo, Italy; antonella.danneo@unipa.it; 6 Department of Biological, Chemical and Pharmaceutical Sciences and Technologies-STEBICEF, University of Palermo, Viale delle Scienze Ed. 16-17, 90128 Palermo, Italy; giuseppe.gallo@unipa.it; 7 NBFC—National Biodiversity Future Center, 90133 Palermo, Italy

**Keywords:** *Lavandula multifida*, phytochemistry, ethnopharmacology, bioactive compounds, mediterranean, biological activities

## Abstract

The genus *Lavandula* represents one of the most valuable aromatic and medicinal plants, holding significant economic importance in the pharmaceutical, food, perfumery, and cosmetics industries. Among them, *L. multifida* is a traditionally used medicinal plant in the Mediterranean region. This work provides a comprehensive review of *L. multifida*, focusing on its traditional uses, phytochemistry, and pharmacological properties. Unlike conventional lavenders, its essential oil is dominated by phenolic monoterpenes, principally carvacrol, alongside significant concentrations of β-bisabolene, 1,8-cineole, and camphor. This distinct phytochemical profile is further complemented by a rich range of non-volatile constituents, including flavonoids, phenolic acids, and triterpenoids. Pharmacological investigations have validated its broad-spectrum antimicrobial activity, demonstrating efficacy against multidrug-resistant bacterial strains and fungal pathogens through mechanisms such as membrane disruption, metabolic interference, and quorum sensing inhibition. Furthermore, the species exhibits significant antioxidant and anti-inflammatory properties, mediated primarily through radical scavenging, cyclooxygenase inhibition, and cytokine modulation. Owing to its distinct chemistry, specific traditional uses for respiratory and digestive ailments, limited endemic habitat, and underexplored status, *L. multifida* presents a promising candidate for future research with high potential for novel drug discovery, particularly in antiparasitic and respiratory therapies. This review concludes by identifying key research priorities for *L. multifida*, including a detailed analysis of its non-volatile compounds, mechanistic elucidation, toxicological assessments, and standardization of extracts. Addressing these gaps is essential to validate its traditional applications and advance its development into evidence-based phytomedicines, adjuvant therapies, and natural agrochemicals.

## 1. Introduction

Since ancient times, humanity has relied on endemic vegetation for sustenance, clothing, shelter, and other essential needs. Across early civilizations, such as those of Egypt, China, and Greece, medicinal plants were pivotal in disease prevention, treatment, and disinfectants [1,2]. Today, these plants remain integral to healthcare systems worldwide, with widespread use in both developed and developing nations [3]. According to the World Health Organization (WHO), approximately 80% of the global population depends on herbal remedies for primary healthcare [4]. Over the past few decades, the demand for plant-derived resources has surged, with an annual growth rate of 8–15% in Europe, Asia, and North America [5]. This rise reflects a growing recognition of medicinal plants’ potential in managing a broad spectrum of diseases, including noncommunicable conditions like diabetes and cancer, as well as infectious diseases such as HIV, tuberculosis, and influenza [6].

The Mediterranean region boasts one of the highest levels of biodiversity on the planet. Despite covering only 1.6% of the Earth’s surface, it harbors approximately 10% of the world’s plant species, with around 25,000 documented species, many of which are endemic [7]. Recent ethnobotanical studies indicate that traditional knowledge of medicinal plants persists throughout the Mediterranean basin, particularly among older generations [8,9,10,11,12]. This knowledge could play a crucial role in the discovery and development of novel phytopharmaceuticals [13]. An estimated 2300 plant and fungal taxa are utilized in the region for various purposes [14]. Among plants, species belonging to the Lamiaceae and Asteraceae families are the most widely used for medicinal applications [15]. Several Mediterranean aromatic and medicinal plants, including lavender (*Lavandula* spp.) [16,17], thyme (*Thymus* spp.) [18,19], rosemary (*Rosmarinus officinalis*) [20,21], spearmint (*Mentha spicata*) [22,23], wormwood (*Artemisia* spp.) [24,25], and marigold (*Calendula officinalis*) [26,27], have been extensively studied for their pharmacological properties.

The genus *Lavandula* L. (Lamiaceae) comprises approximately 40 species, 78 natural intraspecific taxa (including subspecies and varieties), and over 400 cultivated varieties [28]. These plants range from having ephemeral to perennial growth habits and from being small shrubs to dwarf shrubs, with their native distribution centered in the Mediterranean region where they typically inhabit rocky, calcareous soils [29]. Lavenders represent some of the most valuable aromatic and medicinal plants, holding significant economic importance across multiple industries including pharmaceuticals, food flavoring, perfumery, cosmetics, and aromatherapy [30]. Among the numerous taxa, *L. angustifolia*, *L. latifolia*, *L. stoechas*, and the hybrid *L.* × *intermedia* are particularly noteworthy for their commercial significance [31]. Phytochemical investigations have revealed that *Lavandula* species produce diverse secondary metabolites, predominantly monoterpenes and sesquiterpenes in their essential oils, along with triterpenoids and phenolic compounds. Extensive pharmacological studies have demonstrated that these species exhibit a broad spectrum of bioactive properties, including antimicrobial, antifungal, antioxidant, anti-inflammatory, anticancer, antimutagenic, sedative, insecticidal, and larvicidal activities [32].

*Lavandula multifida* L. (commonly known as Egyptian lavender or fern leaf lavender) is a semi-evergreen perennial shrub native to the Mediterranean region, where it grows spontaneously in calcareous soils under hot and dry climatic conditions [33]. This species holds particular botanical significance as the only representative of the *Pterostoechas* section found in the Mediterranean region [34] and one of six *Lavandula* species present in the Algerian flora [35]. Ethnobotanical studies confirm that *L. multifida* is widely recognized and utilized as a medicinal plant throughout Mediterranean countries [9,36]. In Algeria, it has been traditionally employed both as a therapeutic agent for various ailments and as a culinary herb [37,38]. In Morocco and Portugal, it is used to treat ailments of the respiratory, digestive, nervous, and renal systems, and to heal wounds [39,40]. Spanish traditional uses include the treatment of anorexia, fatigue, and digestive problems [41]. In Italy, the plant is known for its rarity and is classified as critically endangered [42]. Among lavender species, *L. multifida* has received considerable scientific attention regarding its phytochemical profile and biological activities. The majority of research has focused on characterizing its essential oil composition and evaluating its antimicrobial [43,44] and antioxidant properties [45,46]. However, relatively few studies have systematically analyzed the chemical constituents of its organic extracts or explored other potential pharmacological activities.

*Lavandula multifida* L. lacks the comprehensive scientific scrutiny afforded to other medicinal plants and congeneric species. In North Africa (Tunisia) and Italy, it is now classified as endangered and is cultivated in protected areas to mitigate its population decline, a threat primarily driven by anthropogenic habitat disturbance [33,43]. This species is notably physiologically adapted to arid conditions [47] and exhibits exceptional salinity tolerance within the Lamiaceae family, a key trait required for its persistence in xeric and saline environments [48]. It is distinguished by a particular phytochemical profile dominated by antimicrobial phenols such as thymol and carvacrol, which contribute to wound healing and potential antiparasitic effects, as well as by beta-bisabolene, which adds balsamic notes and may offer anti-inflammatory benefits [49,50]. Additionally, 1,8-cineole imparts a strong medicinal aroma and supports its traditional use as a respiratory expectorant [51]. This combination aligns the plant’s chemistry and bioactivity more with oregano or thyme, underpinning its ethnopharmacological uses for infections, respiratory issues, and digestive complaints. Furthermore, its traditional application in treating some diverse ailments such as inflammatory conditions, diabetes, and wound healing is increasingly supported by preliminary phytochemical and pharmacological evidence.

To the best of our knowledge, no comprehensive review has been published to date on the traditional uses, chemical composition, and pharmacological properties of *Lavandula multifida*. This species is chemically unique, ecologically significant and a conservation priority, yet scientifically underexplored despite its rich traditional use. Therefore, this review aims to consolidate the existing knowledge on its ethnomedicinal applications, phytochemical profile, and biological activities, thereby providing a foundational resource for future research.

## 2. Methodology

In this literature review, a comprehensive search methodology was followed to ensure the broad coverage of significant scientific publications. The primary search was conducted across major databases: Scopus, PubMed/MEDLINE, ScienceDirect, SpringerLink, Wiley Online Library, and JSTOR. Additionally, the search included the search engine Google Scholar and the academic platforms ResearchGate, Academia.edu, and Semantic Scholar. The search keywords were ‘*Lavandula multifida*’, ‘Egyptian lavender’, ‘traditional use’, ‘biological activity’, ‘pharmacology’, ‘chemical composition’, ‘essential oil’, ‘extract’, ‘antioxidant activity’, ‘antimicrobial activity’, and ‘phytochemistry’, linked by logical operators (AND, OR). Additionally, the search incorporated ethnobotanical studies on the Mediterranean area and essential oil components, and biological properties of particular molecules. It was also limited to articles published in English or French. This process produced a core set of references that form the basis of this review. Their findings directly shaped its thematic structure.

## 3. Botanical Characterization and Geographical Distribution

### 3.1. Taxonomy

The Lamiaceae family (also known as Labiatae) represents one of the most important groups of flowering plants, comprising approximately 236 genera and 6900–7200 species [52,53,54,55]. Within its seven recognized subfamilies, the Nepetoideae subfamily includes the genus *Lavandula* L. [32]. This genus encompasses 40 species, 78 intraspecific taxa (including subspecies and varieties) and natural hybrids, along with more than 400 cultivated varieties [28]. Taxonomically, *Lavandula* is divided into three subgenera (*Lavandula*, *Fabricia*, and *Sabaudia*) and further classified into eight sections (*Lavandula*, *Dentatae*, *Stoechas*, *Pterostoechas*, Subnudae, *Chaetostachys*, *Hasikenses*, and *Sabaudia*). Of particular interest, *L. multifida* belongs to the *Pterostoechas* section within the *Fabricia* subgenus [32], making it taxonomically distinct among Mediterranean lavender species.

### 3.2. Taxonomic Classification

The complete taxonomic classification of *Lavandula multifida* L. is as follows [56]:
KingdomPlantaeDivisionStreptophytaClassEquisetopsidaSubclassMagnoliidaeOrderLamialesFamilyLamiaceaeGenusLavandulaSpecies*Lavandula multifida* L.

The World Flora Online (WFO) [57] database lists the following synonyms for *Lavandula multifida* L.:

*Lavandula multipartita* Christm.

*Lavandula pinnatifida* Webb

*Lavandula multifida* var. *heterotricha* Wild

*Lavandula multifida* var. *homotricha* Wild

*Lavandula multifida* var. *intermedia* Ball

*Lavandula multifida* var. monostachya Lundmark

*Lavandula multifida* f. *albiflora* H.Lindb.

*Lavandula multifida* f. *glabrifolia* Pau

*Lavandula multifida* f. *pallescens* Maire

### 3.3. Botanical Description

**Figure 1:**
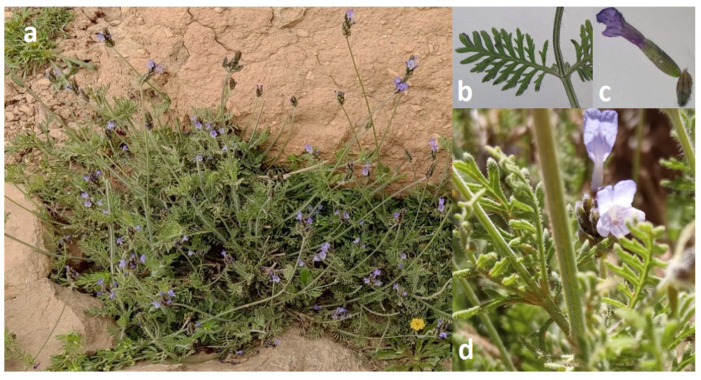
*Lavandula multifida* plant from M’sila (Algeria): (**a**) Plant tuft; (**b**) Leaf; (**c**) Flower with bract; (**d**) Inflorescence. Original photographs—Hendel N.

*Lavandula multifida* L. is a semi-evergreen perennial shrub with a diploid genome, typically growing to heights of 30–100 cm (Figure 1). This species is morphologically characterized by its triangular–pinnatisect leaves and vibrant purple–blue flowers measuring 10–12 mm in length. The stems bear both long simple white trichomes and distinctive short, branched hairs, while the inflorescence features a basally branched stalk supporting characteristically twisted floral spikes. The corolla displays a bicolored pattern, with violet lower lobes gradually transitioning to blue–violet upper lobes marked by darker nectar guides. The elliptic bracts exhibit sharply acute apices and typically show three prominent dark veins, while the calyx presents a clearly deltoid-shaped upper medial lobe [34,58,59]. Among *Lavandula* species, *L. multifida* is particularly notable for its unique fern-like foliage, twisted flower spikes, and strong aromatic profile that differs significantly from the characteristic scent of common lavender [35].

### 3.4. Geographical Distribution

**Figure 2:**
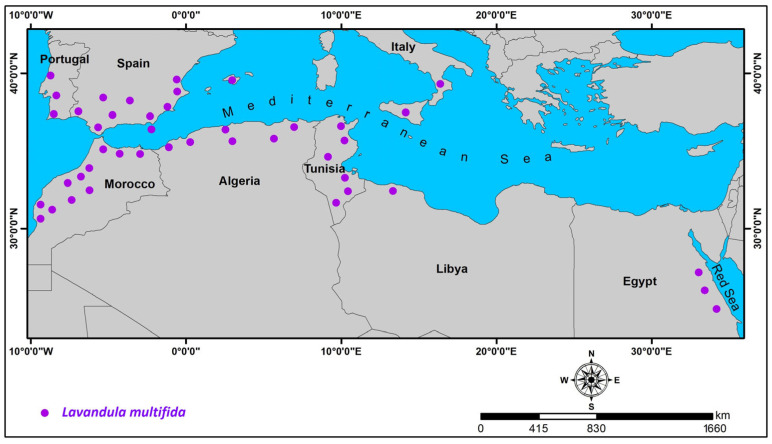
Geographic Distribution of *L. multifida* (generated using the referenced data).

*Lavandula multifida* L. is native to the southwestern Mediterranean Basin, with a natural distribution spanning North Africa (Morocco, Algeria, Tunisia, Libya, and Egypt) and southern Europe (Italy, Spain, and Portugal) [60,61] (Figure 2). As the only representative of the *Pterostoechas* section abundant in this region [35], it exhibits distinct distribution patterns across its range.

In Europe, the species shows contrasting abundance patterns. Italy hosts only scattered populations in Calabria and Sicily, where it ranks among the nation’s rarest flora, typically growing in garrigue formations on immature limestone soils at 10–200 m elevation under arid conditions. Conversely, on the Iberian Peninsula, *L. multifida* demonstrates wider ecological tolerance, occurring from coastal areas up to 800 m altitude. Spanish populations extend continuously from Huelva to Valencia [61], while Portuguese specimens thrive naturally in southern locales including Sesimbra, Arrábida, and Mértola [34].

North African distributions reveal further ecological specialization. Moroccan populations predominantly occupy pre-Saharan zones (800–2000 m elevation), favoring calcareous substrates and temporary river margins [53]. Tunisian specimens form fragmented populations at 100–790 m elevation in central and southern regions, typically colonizing marl or limestone outcrops, exposed ridges, and degraded *Pinus halepensis* L. and *Juniperus phoenicea* L. forest habitats [62]. Algerian distributions concentrate in northern territories, while Libyan occurrences are focused in Tripolitania. Egyptian populations are restricted to Red Sea coastal areas [60].

## 4. Traditional Uses

Lavender species (*Lavandula* spp.) have been employed medicinally since the Middle Ages for their diverse therapeutic properties, including antibacterial, antifungal, antidepressant, sedative, and carminative effects, as well as for treating insect stings, burns, pain, tremors, epilepsy, and migraines [63]. *L. multifida* emerges as one of the most extensively utilized lavender species in Mediterranean traditional medicine [37] (Table 1), with applications varying regionally across its native range. In the Maghreb countries, Algerian traditional medicine employs it against influenza, hypertension, and cancer, while also using it as a sedative, stomachic, antispasmodic agent, and culinary tea ingredient [38,39]. Moroccan practitioners value it particularly highly, prescribing it for over twenty distinct conditions ranging from rheumatoid disorders and gastrointestinal ailments to microbial infections, wound healing, hemorrhages, uterine inflammation, abscesses, asthma, and cystitis [64], while Tunisian tradition utilizes leaf decoctions as hypotensive, emmenagogue, and antidiabetic remedies [65]. Iberian applications include its use in Portugal for respiratory disorders, vertigo, skin conditions, and as a digestive tonic with soporific and carminative properties [39], alongside the Spanish employment of aerial part infusions for digestive complaints [66]. Despite this widespread traditional use throughout the Mediterranean basin, a substantial proportion of these therapeutic applications currently lack empirical validation through modern pharmacological studies.

## 5. Phytochemicals

A comprehensive understanding of bioactive secondary metabolites is essential for optimizing the utilization of medicinal plants in pharmaceutical, agricultural, and culinary applications [70]. The genus *Lavandula* represents a significant reservoir of diverse phytochemicals, though research has predominantly focused on volatile compounds while largely neglecting non-volatile secondary metabolites [32,71]. Phytochemical investigations have identified three major classes of compounds in *Lavandula* species: (1) essential oil components (primarily monoterpene and sesquiterpene derivatives), (2) triterpenoids [32], and (3) phenolic compounds including protocatechuic, caffeic, and rosmarinic acids [71]. Extensive essential oil analyses of *L. angustifolia*, *L.* × *intermedia*, *L. stoechas*, *L. dentata*, *L. viridis*, *L. latifolia*, *L. luisieri*, and *L. multifida* have consistently identified linalool, 1,8-cineole, and camphor as major constituents [30]. Similar to other lavender species, *L. multifida* has been primarily studied for its essential oil composition, with organic extracts receiving comparatively less attention. Table 2 presents the phytochemical compounds isolated from *L. multifida*. Preliminary phytochemical screening of *L. multifida* has consistently revealed a wide spectrum of secondary metabolites, including terpenoids, flavonoids, tannins, mucilage, sterols, and saponins [46,72]. The quantification of its polyphenol and flavonoid contents via colorimetric assays confirms a notable richness in these compounds; however, reported values exhibit considerable variation depending on the extraction conditions and plant origin. For example, the accelerated extraction of Spanish *L. multifida* methanolic extracts yielded a total phenolic content of 179 mg GAE/g extract [73]; meanwhile, aqueous decoctions of Moroccan material produced notably higher levels, with total phenolic and flavonoid contents of 199.16 ± 11.20 mg GAE/g and 142.55 ± 1.66 mg RE/g extract, respectively [74]. Algerian investigations on crude methanolic extracts revealed a pronounced enrichment in both polyphenols and flavonoids, with the ethyl acetate fraction reaching 462.23 ± 11.74 µg GAE/mg extract and 125.90 ± 0.16 µg QE/mg extract, respectively [75]. Algerian specimens contain seven phenolic compounds (notably apigenin, rutin, and coumarin) and thirteen fatty acids (predominantly oleic, linoleic, and stearic acids) [76], while Moroccan populations yield four triterpenes (maslinic, oleanolic, and ursolic acids), five diterpenes, and two monoterpenes (carvacrol and carvacrol-3-glucoside) from ethanol extracts [77]. Spanish samples demonstrate particular phytochemical complexity, with methanolic extracts containing 65 identified compounds, including pentacyclic triterpenes (maslinic, madecassic, quillaic, glycyrrhetinic, medicagenic, and asiatic acids) and diverse flavonoids (phloretin xyloglucoside, hesperetin, quercetin glucoside, rutin, and various glucosides) [73]. Additional studies have identified carvacrol and nine flavone derivatives (including vitexin, apigenin, and multiple 7-O-glucoside compounds) in leaf methanolic extracts [42], further highlighting the species’ chemical diversity and potential pharmacological value.

### Chemical Constituents of L. multifida Essential Oils

The essential oil yield of *L. multifida* ranges from 0.09% to 2.4% (*w*/*w*) of dry weight and can be extracted from leaves, stems, and flowers using various methods, with hydrodistillation being the most common technique, followed by steam distillation and headspace solid-phase microextraction (HS-SPME) [78,79]. Analyses of essential oils from different Mediterranean populations (Morocco, Tunisia, Algeria, and Portugal) consistently reveal a characteristic chemical profile dominated by oxygenated monoterpenes (33.70–92.58%), with lower proportions of monoterpene hydrocarbons (0.25–41.4%), sesquiterpene hydrocarbons (0.14–35.6%), and oxygenated sesquiterpenes (0.10–11.81%) [80,81,82,83].

**Figure 3:**
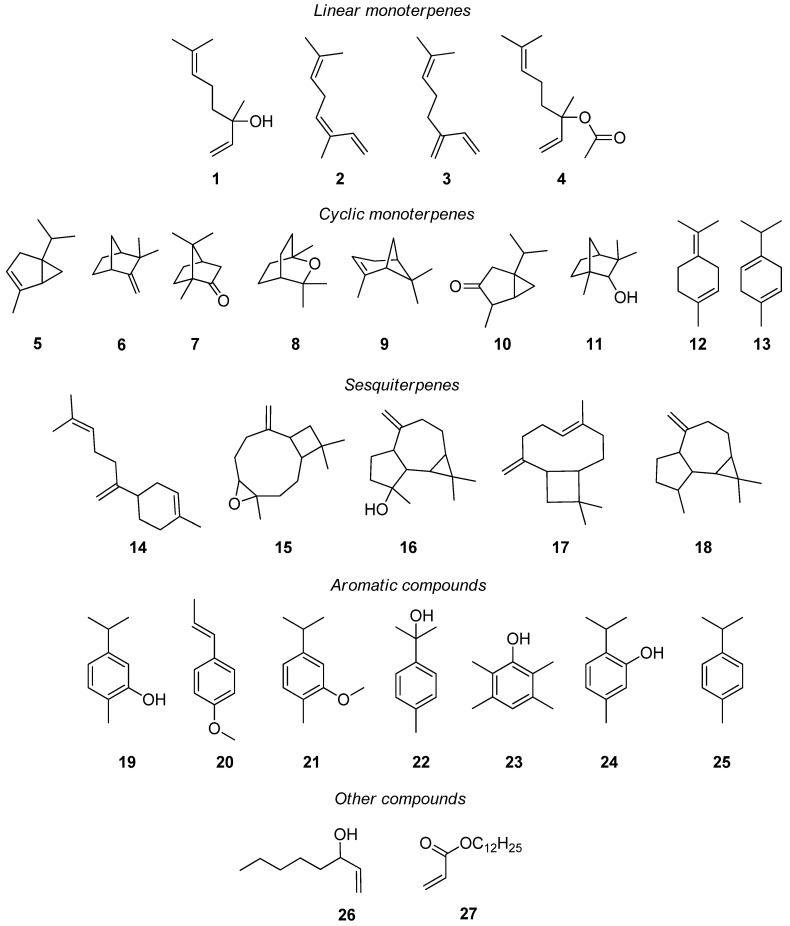
Chemical structures of major compounds in *L. multifida* essential oil. Linalool **1**, cis-β-ocimene **2**, myrcene **3**, linalyl acetate **4**, α-thujene **5**, camphene **6**, camphor **7**, 1,8-cineole **8**, α-pinene **9**, α-thujone **10**, fenchol **11**, terpinolene **12**, γ-terpinene **13**, β-bisabolene **14**, caryophyllene oxide **15**, spathulenol **16**, β-caryophyllene **17**, aromadendrene **18**, carvacrol **19**, anethole **20**, carvacrol methyl ether **21**, p-cymen-8-ol **22**, durenol **23**, thymol **24**, p-cymene **25**, 1-octen-3-ol **26**, acrylic acid dodecyl ester **27** [84].

The chemical structures of major compounds in *L. multifida* essential oil are reported in Figure 3.

Notably, carvacrol **19** emerges as the predominant constituent across most studies, typically representing 27.77–66.2% of the total essential oil composition (Table 3).

An analysis of Algerian *L. multifida* essential oils by Saadi et al. [78] demonstrated significant compositional differences between plant parts, with inflorescence oils dominated by carvacrol **19** (61.73%), linalool **1** (5.69%), and 1-octen-3-ol **26** (3%), while leaf oils primarily contained carvacrol **19** (50.92%), anethole **20** (17.37%; reported for the first time in this species), β-bisabolene **14** (5.81%), and linalool **1** (3.42%). Complementary research by Khadir et al. [79] on aerial parts across three phenological stages revealed dynamic changes in major components: carvacrol **19** peaked during full inflorescence (57.1%) compared to before (29.7%) and after (27.5%) flowering, while β-bisabolene **14** showed an inverse pattern (37.8%, 25.2%, and 38.4%, respectively). Caryophyllene oxide **15** (7.5%, 3.7%, 6.8%) and spathulenol **16** (2.9%, 3.4%, 6.2%) demonstrated a consistent presence throughout development, with their relative concentrations varying according to growth stage.

A comparative analysis of *L. multifida* essential oils from two Portuguese regions (Sesimbra/Arrábida and Mértola) revealed a consistent chemical profile dominated by carvacrol **19** (42.8% and 41.5%, respectively), cis-β-ocimene **2** (27.4% and 27.0%), myrcene **3** (5.7% and 5.5%), and β-bisabolene **14** (5.6% and 5.0%), marking the first reported occurrence of cis-β-ocimene **2** in this species [34]. Recent follow-up studies in Portugal confirmed this chemical pattern, with the essential oil composition showing substantial qualitative similarity, though with some quantitative variation: carvacrol **19** (46.4%), cis-β-ocimene **2** (12.7%), β-bisabolene **14** (10.1%), and myrcene **3** (5.9%) [80]. These findings demonstrate both the consistent predominance of carvacrol across Portuguese populations and the natural variability in secondary compound concentrations between different growing regions and studies.

Tunisian *L. multifida* essential oils predominantly feature carvacrol **1** as their major constituent, though significant chemotypic variation exists across populations. Chograni et al. [62] analyzed twelve wild populations and identified carvacrol **19** (31.81%), β-bisabolene **14** (14.89%), and acrylic acid dodecyl ester **27** (11.43%) as principal components, while Messaoud et al. [81] reported higher concentrations of carvacrol **19** (65.1%) accompanied by β-bisabolene **14** (24.7%) and β-caryophyllene **17** (2.4%). However, distinct chemotypes have emerged under different growing conditions: Msaada et al. [55] documented a linalool **1**-dominant profile (50.05%) in cultivated specimens, with camphene **6** (10.06%), linalyl acetate **4** (7.30%), and α-thujene **5** (3.83%) as co-dominant compounds. More recently, Tofah et al. [44] identified a novel chemotype characterized by camphor **7** (15.68%), 1,8-cineole **8** (14.14%), and α-pinene **9** (13.82%) as major constituents. These findings collectively demonstrate substantial intraspecific chemical diversity in Tunisian *L. multifida*, influenced by both genetic factors and cultivation conditions.

The essential oil composition of Moroccan *L. multifida* has been extensively studied, revealing a complex chemical profile dominated by monoterpenes, with carvacrol **19** as the predominant compound. Multiple studies have identified consistent major components including spathulenol **16**, carvacrol methyl ether **21**, p-cymen-8-ol **22**, caryophyllene oxide **15**, β-bisabolene **14**, dodecyl acrylate **27**, linalool **1**, α-thujone **10**, fenchol **11**, and terpinolene **12** [44,45,82,83,85]. However, significant chemotypic variation exists among Moroccan populations: Soro et al. [72] reported an unusual durenol **23**-dominant chemotype (89.97%) accompanied by caryophyllene oxide **15** (2.43%), spathulenol **16** (1.83%), and aromadendrene **18** (1.68%), while El Rhaffari et al. [64] documented a thymol **24**-rich profile (32%) with carvacrol **19** (27.77%), p-cymene **25** (15.72%), and γ-terpinene **13** (9.54%). Znini et al. [53] further demonstrated that extraction methods influence the quantitative composition, with both HS-SPME and hydrodistillation yielding oils rich in carvacrol **19** (65.6% and 57.9%, respectively), carvacrol methyl ether **21** (4.6% and 7.6%), p-cymen-8-ol **22** (4.8% and 3.9%), and spathulenol **16** (8.6% and 3.8%). These findings collectively highlight both the consistent qualitative patterns and remarkable quantitative variability in Moroccan *L. multifida* essential oils across different populations and extraction techniques.

Awad et al. [86] also reported an Egyptian chemotype of *L. multifida* that was distinct from carvacrol-dominated profiles. Its essential oil was primarily composed of 1,8-cineole **8** (39.84%), followed by camphor **7** (18.86%), and α-pinene **9** (8.86%).

The observed variations in *L. multifida* essential oil composition can be attributed to multiple interrelated factors, including (1) harvesting time, (2) plant varieties, (3) growth stage, (4) plant parts utilized, (5) extraction methodologies, (6) analytical techniques, (7) cultivation conditions, (8) environmental influences, and (9) genetic factors [43,45,53,55,78,79,84]. Additionally, these compositional differences may arise from the dynamic bioconversion of certain constituents into their isomers during plant development or post-harvest processing [43,45,53]. The chemical complexity and variability of *L. multifida* essential oils are visually represented in Figure 3 [86], which highlights the major constituents and their structural relationships. This multifaceted chemical diversity underscores the importance of standardizing collection and analysis protocols when studying *L. multifida* essential oils for research or commercial applications.

**Table 3:** Major constituents of volatile oils from *L. multifida*.

Country of Origin	Plant Part	Extraction Method	Analytical Method	Oil (%,*v*/*w*)	Nb. of Compounds/Oil%	Major Compounds	Compound Category	Ref.
Algeria (Chlef—northern Algeria)	Inflorescences	SD	GC-FID; GC-MS	1%	29/(92%)	carvacrol (61.73%) linalool (5.69%) 1-octen-3-ol (3%)	OM (74.68%) MH (1.84%) OS (0.64%) SH (2.82%)	[42]
	Leaves	SD	GC-FID; GC-MS	0.8%	43/(96.25%)	carvacrol (50.92%) anethole (17.37%) β-bisabolene (5.81%)	OM (58.25%) MH (1.47%) OS (0.59%) SH (8.02%)	
Algeria (Tlemcen—North-West of Algeria)	Aerial parts	HD	GC-FID; GC-MS	0.2%	23/(98.4%)	carvacrol (57.1%) β-bisabolene (25.2%) caryophyllene oxide (3.7%) spathulenol (3.4%)	OM (60.1%) MH (3.8%) OS (7.1%) SH (27.4%)	[78]
Egypt	Leaves	HD	GC-MS	NI	23/(99.72%)	1,8-cineole (39.84%) camphor (18.86%) α-pinene (8.86%)	OM (65.52%) MH (23.18%) OS (3.40%) SH (6.10%)	[86]
Morocco (Errachidia- South-Eastern Morocco)	Aerial parts	HD	GC-FID; GC-MS	0.7%	34/(96.5%)	carvacrol (66.2%) spathulenol (4.9%) p-cymene-8-ol (4.2%) caryophyllene oxide (2.7%) terpinolene (2.6%)	OM (73.8%) MH (8.2%) OS (7.7%) SH (1.1%)	[45]
Morocco (Tetuan-North-western Morocco)	Aerial parts	HD	GC-FID; GC-MS	0.097%	34/(95.25%)	carvacrol (47.62%) β-bisabolene (9.01%) dodecyl acrylate (8.37%) linalol (7.42%)	OM (62.51%) MH (11.09%) OS (9.27%) SH (12.38%)	[81]
Morocco (Errachidia- South-Eastern Morocco)	Leaves and flowers	HD	GC-FID; GC-MS	2.4%	28/(86.2 ± 86.8%)	carvacrol (57.9 ± 59.0%) carvacrol methyl ether (7.0 ± 7.6%) p-cymen-8-ol (3.9 ± 4.7%)	OM (72.9 ± 81.1%) MH (1.8 ± 8.2%) OS (2.4 ± 3.0%) SH (0.2 ± 1.2%)	[82]
Morocco Rabat- North- western Morocco	Leaves and stems	HD	GC-FID; GC-MS	0.46%	20/(97.8%)	carvacrol (44.3%) β-bisabolene (31.9%) careophylene oxide (5.8%) fenchol (3.2%)	OM (48.7%) MH (6.8%) OS (6.2%) SH (35.6%)	[44]
Morocco (Anti Atlas region)	Aerial parts	SD	GC; GC-MS	2.01%	22/(99.59%)	durenol (89.97%) caryophyllene oxide (2.43%) sphatulenol (1.83%) aromadendrene (1.68%) carvacrol methyl ether (1%)	OM (92.58%) MH (0.25%) OS (0.6%) SH (6.48%)	[72]
Morocco (Errachidia- South-Eastern Morocco)	Leaves and flowers	SD	GC-FID; GC-MS	1.2%	39/(99.76%)	thymol (32.00%) carvacrol (27.77%) p-cymene (15.72%) γ-terpinene (9.54%)	OM (81.49%) MH (2.75%) OS (11.81%) SH (0.23%)	[64]
Morocco (Errachidia- South-Eastern Morocco)	Aerial parts	HS-SPME HD	GC-FID; GC-MS GC-FID; GC-MS	NI* 2.4%	21/(90.2%) 29/(90.6%)	carvacrol (65.6%) spathulenol (8.6%) p-cymene-8-ol (4.8%) carvacrol methyl ether (4.6%) carvacrol (57.9%) carvacrol methyl ether (7.6%) p-cymene-8-ol (3.9%) spathulenol (3.8%)	OM (75.8%) MH (3.1%) OS (8.7%) SH (1.7%) OM (72.9%) MH (8.2%) OS (6.6%) SH (1%)	[53,83]
Portugal (Sesimbra/Arrábida and Mértola—south of Portugal)	Aerial parts	HD	GC; GC-MS	NI	33/(97.9%)	carvacrol (42.8% and 41.5%) cis-β-ocimene (27.4% and 27.0%) myrcene (5.7% and 5.5%) β-bisabolene (5.6% and 5.0%)	OM (43.2% and 41.8%) MH (41.4% and 38.5%) OS (1.5% and1.8%) SH (10.8%and 10.5%)	[34]
Portugal Sesimbra—south of Portugal)	Aerial parts	HD	GC; GC–MS	NI	31/(95.2%)	carvacrol (46.4%) cis-β-ocimene (12.7%) β-bisabolene (10.1%) myrcene (5.9%)	OM (48.8%) MH (25.1%) OS (3.7%) SH (14.9%)	[79]
Tunisia	Leaves	HD	GC-FID; GC-MS	NI	36/(83.48%)	carvacrol (31.81%) β -bisabolene (14.89%) acrylic acid dodecanyl ester (11.43%)	OM (33.70%) MH (15.76%) OS (6.38%) SH (14.89%)	[62]
Tunisia (Sidi Bouzid- Central West of Tunisia)	Stems leaves	HD	GC-FID; GC-MS	0.26%	29/(98.3%)	carvacrol (65.1%) β-bisabolene (24.7%) β-caryophyllene (2.4%) myrcene (5.7% and 5.5%)	OM (65.7%) MH (1.5%) OS (1.0%) SH (30.1%)	[80]
Tunisia (Grombalia—North-Eastern Tunisia)	Aerial parts	HD	GC-FID; GC-MS	1.62%	52/(98.21%)	linalool (50.05 ± 6.52%) camphene (10.06 ± 1.21%) linalyl acetate (7.30 ± 0.65%) α-thujene (3.83 ± 0.41%)	OM (71.40%) MH (20.70%) OS (0.10%) SH (5.27%)	[55]
Tunisia (Agareb—North-Eastern Tunisia)	Aerial parts	HD	GC-MS	NI	58/(98.98)	camphor (15.68%) 1,8-cineole (14.15%) α-pinene (13.82%) linalool (9%)	OM (45.98%) MH (11.79%) OS (7.37%) SH (0.14%)	[43]

Notes: NI (Not indicated); OM (Oxygenated monoterpenes); MH (Monoterpene hydrocarbons); OS (Oxygenated sesquiterpenes); SH (Sesquiterpene hydrocarbons); HD (Hydrodistillation); SD (Steam distillation).

## 6. Pharmacological Activities

The diverse pharmacological properties of *Lavandula* species are primarily attributed to their essential oil constituents, though non-volatile phenolic compounds also contribute significantly to their bioactivity [87]. Extensive research has demonstrated that *Lavandula* species exhibit a broad spectrum of biological activities, including antibacterial [88,89,90], antifungal [31,91], antioxidant [92,93], anti-inflammatory [94,95,96], anticancer [97,98], neuroprotective [99,100,101], antispasmodic, anxiolytic, analgesic, antiseptic, and sedative effects [102,103,104], along with notable antiparasitic [105,106] and insecticidal properties [107,108] (Table 4). As an important ethnobotanical remedy, *L. multifida* has been traditionally used to treat various infections and diseases. While current pharmacological investigations have predominantly focused on its essential oil’s antibacterial and antioxidant properties, other potential biological activities of this species remain substantially underexplored, despite its widespread traditional use and promising phytochemical profile.

### 6.1. Antimicrobial Activity

Multiple studies have demonstrated the significant antibacterial potential of *L. multifida* essential oils against various pathogenic bacterial strains. Tofah et al. [43] evaluated its activity against *Escherichia coli* ATCC 8739 and *Staphylococcus aureus* ATCC 6583 using the agar well diffusion method, revealing notable efficacy (MIC = 0.625 μg/mL) with greater sensitivity in *E. coli* (inhibition diameter = 22.6 mm) compared to *S. aureus* (17.8 mm). Elmakaoui et al. [44] analyzed essential oil collected during the flowering stage in northwestern Morocco, reporting carvacrol (44.3%) as the major constituent, followed by β-bisabolene (31.9%) and caryophyllene oxide (5.8%). They expanded this investigation through disk diffusion testing against five pathogenic strains (*Staphylococcus epidermidis*, *S. aureus*, *Acinetobacter baumannii*, *Klebsiella pneumoniae*, and *E. coli*), with *S. aureus* showing the highest susceptibility. Antibacterial activity was confirmed against the same strains with MIC values ranging from 2 to 4 μg/mL [44]. Further supporting these findings, Douhri et al. [82] reported broad-spectrum inhibition against both Gram-positive (*S. aureus*, *Bacillus subtilis*, *Listeria monocytogenes*) and Gram-negative (*Proteus mirabilis*, *P. vulgaris*, *E. coli*) bacteria, particularly noting strong activity against *S. aureus*, *B. subtilis*, and *P. mirabilis*.

*Lavandula multifida* has demonstrated remarkable efficacy against multidrug-resistant bacterial strains, particularly those associated with nosocomial infections. Soro et al. [72] reported the potent antibacterial activity of its essential oil against multidrug-resistant *Escherichia coli* (inhibition diameter: 37.3 ± 4.4 mm), *Klebsiella pneumonia* (36.3 ± 2 mm), and *Pseudomonas aeruginosa*, with the highest sensitivity observed in the first two pathogens. The essential oils from the aerial parts of *L. multifida* were analyzed at three developmental stages [79], with the essential oil yield varying depending on the phenological phase. The highest content (0.2% w/w) was recorded at the full-inflorescence stage. GC and GC–MS analyses consistently identified carvacrol (27.5–57%), β-bisabolene (25.2–38.4%), and caryophyllene oxide (3.5–7.5%) as the main constituents. They further demonstrated superior anti-methicillin-resistant *S. aureus* (MRSA) activity compared to other *Lavandula* species, with inhibition zones ranging from 14 to 27 mm for the essential oil, while the ethanol extract showed reduced effectiveness. In particular, *L. multifida* essential oil has shown a MIC < 0.1 μL/mL [79].

Interestingly, similar findings were confirmed in samples rich in carvacrol collected in Meknes (Morocco), with MICs of 2 and 12 μg/mL against *E. coli* and *S. aureus*, respectively [109].

**Table 4:** A summary of the documented pharmacological activities of *L. multifida*.

Activity	Test	Extracts/Compounds	Targets; Model	Effects; Key Findings	Ref.
Antibacterial	In vitro	EO; isolated camphor	*E. coli*, *S. aureus*	- EO: Strong antibacterial activity; 22.6 mm (*E. coli*), 17.8 mm (*S. aureus*); effect due to synergy of multiple components. - Camphor: Weaker (13.2 mm *E. coli*, 14.1 mm *S. aureus* at 2.5 µg/mL).	[43]
		EO	*S. aureus*, *S. epidermidis*, *E. coli*, *K. pneumoniae*, *A. baumannii*	- Strong antibacterial activity (IZ 9–20 mm; MIC 0.5–4 µg/mL); *S. aureus* most sensitive. - Effect linked to carvacrol and β-bisabolene, with possible synergistic action.	[44]
		EO	*S. aureus*, *B. subtilis*, *L. innocua*, *L. monocytogenes*, *E. coli*, *P. vulgaris*, *P. mirabilis*, *P. aeruginosa*	- Moderate antibacterial activity (IZ 8.5–16 mm; MIC 1–>4% *v*/*v*); *S. aureus* most sensitive. - Effect linked to carvacrol and β-bisabolene, with possible synergistic action.	[82]
		EO	*E. coli*, *K. pneumoniae*, *P. aeruginosa*	- Strong antibacterial activity (IZ 10.6–37.3 mm; MIC 0.6–4.8 µL/mL). - Most sensitive: *E. coli*, *K. pneumoniae*; *P. aeruginosa* was less sensitive. - Effect linked to durenol with possible synergy from other compounds.	[72]
		EO; ethanol extract	*Methicillin-resistant S. aureus* strains (MRSA), *Methicillin-sensitive S. aureus* strains (MSSA)	- EO: Strong anti-MRSA activity (inhibition zones 14–27 mm; MIC 0.6–5 µL/mL), with MSSA and some MRSA strains being most sensitive. - The ethanol extract was less active. - Effect linked to carvacrol, β-bisabolene and caryophyllene oxide, with possible synergistic action.	[79]
		EO; isolated linalool	*S. epidermidis* (biofilm-forming strain)	- EO: Strong anti-biofilm activity (1.3–1.6 log at 10–30%) and synergized with CHG (~3.3–3.7 log) and CHG+IPA (up to 5.5 log). - Linalool: No significant difference in anti-biofilm effect or in synergy with disinfectants. - Effect linked to linalool.	[110]
Antifungal	In vitro	EO; isolated Carvacrol, cis-β-Ocimene	*Candida* spp., *Cryptococcus neoformans*, dermatophytes (*Trichophyton* spp., *Microsporum* spp., *Epidermophyton floccosum*), *Aspergillus* spp.	- EO: Broad antifungal activity; most active against dermatophytes and *C. neoformans* (MIC 0.16 µL/mL). - Carvacrol more active (MIC 0.04–0.16 µL/mL); cis-β-ocimene less active but contributed to filamentation inhibition.	[34]
		EO	*Alternaria* sp., *P. expansum*, *R. stolonifer*, *B. cinerea*	- Strong antifungal activity; complete inhibition of *R. stolonifer* and *Alternaria* sp. (100 µg/mL). - Effect linked to carvacrol with possible synergy.	[45]
		EO	*Alternaria* sp., *P. expansum*, *R. stolonifer*	- Strong antifungal; MIC 0.25–0.5 µL/mL; fungicidal at 0.5–1 µL/mL. - Effect mainly linked to carvacrol with synergy from other constituents.	[84]
		EO	*C. albicans*, dermatophytes (*E. floccosum*, *M. gypseum*, *M. canis*, *T. mentagrophytes*, *T. interdigitale*, *T. rubrum*)	- Strong antibiofilm activity; inhibited biofilm formation (from 0.32 µL/mL) and disrupted mature biofilms (46% biomass, 49% matrix, 30% viability); most active against *E. floccosum*. - Effect mainly linked to carvacrol with contribution from cis-β-ocimene and β-bisabolene.	[80]
Antioxidant	in vitro; in vivo	Hydro-methanolic extract	Radical scavenging (DPPH, TEAC, ORAC), reducing power (FRAP); oxidative stress model (mice fed a high-fat diet)	- In vitro Strong antioxidant (DPPH IC_50_ = 8.06 µg/mL; FRAP = 2.58 mmol Fe^2+^/g; TEAC = 1.30 mmol Trolox/g; ORAC = 2.08 mmol Trolox/g). - In vivo, reduction in oxidative stress and liver lipid peroxidation (decreased TBARS). - Effect linked to polyphenols, triterpenes, and flavonoids.	[73]
	In vitro	Crude extracts; fractions (dichloromethane, ethyl acetate, butanol)	Radical scavenging (DPPH, Galvinoxyl, ABTS), reducing power (CUPRAC, FRAP, phenanthroline), metal chelation assays.	- Ethyl acetate fraction: Strong antioxidant activity; DPPH (EC_50_ = 12.32 µg/mL, < BHA 5.73); superior in ABTS (4.89 µg/mL), Galvinoxyl (9.60 µg/mL), and reducing power (FRAP, CUPRAC, phenanthroline) - All extracts and fractions weak in metal chelation (>800 µg/mL). - Effect linked to polyphenols and flavonoids.	[75]
		EO	Radical scavenging (DPPH), lipid peroxidation inhibition (β-carotene bleaching)	- Moderate DPPH scavenging activity (IC_50_ = 16.83 µg/mL; BHT = 7.73 µg/mL). - Strong β-carotene inhibition (78.4%; BHT = 86.2%). - Effect linked to carvacrol and phenolics.	[45]
		Aqueous extract	Radical scavenging (DPPH), reducing power (FRAP)	- Strong DPPH scavenging (IC_50_ = 2.6 mg/mL); FRAP = 12.76 mmol Trolox/g. - Effect linked to polyphenols and flavonoids.	[74]
		EO; methanol extract	Radical scavenging (DPPH), reducing power (FRAP), metal chelation	- Methanol extract: Strong antioxidant; DPPH IC_50_ = 19.3 µg/mL (BHT 26.5; Trolox 12.8); FRAP = 377.8 mmol/g; chelation IC_50_ = 0.8 µg/mL. - Essential oil: Weaker antioxidant; DPPH IC_50_ = 201.6 µg/mL; FRAP = 39.1 mmol/g; chelation IC_50_ = 7.9 µg/mL. - Effect linked to polyphenols and carvacrol.	[81]
		Methanolic extract	Radical scavenging (DPPH assay)	- Moderate DPPH scavenging (IC_50_ = 17.36 mg/mL) - Stronger than *L. stoechas*, weaker than *L. dentate*. - Effect linked to polyphenols.	[46]
Anti-inflammatory		Aqueous and ethanol extracts; isolated compounds from ethanol extract	Croton oil-induced ear edema in mice	- Ethanol extract: strong topical anti-inflammatory (17–62% edema reduction, ID_50_ = 510 µg/cm^2^; ~5× weaker than indomethacin); - Aqueous extract: Weaker, active only at high doses (24–33% reduction). - Isolated compounds (ursolic acid, oleanolic acid, maslinic acid) highly active (41–74% reduction) - Effect linked to ethanol-soluble triterpenoids, ursolic acid, oleanolic acid, and maslinic acid.	[77]
	in vivo	Aqueous extract	formaldehyde-induced paw edema in Rats	- Moderate anti-inflammatory (10–44% inhibition of paw edema, 200 mg/kg); weaker than diclofenac. - Effect linked to flavonoid and phenolic constituents.	[75]
Insecticidal	in vitro	EO	*Spodoptera littoralis*, *Agrotis ipsilon*	- Strong toxicity (LC_50_ = 2.35 mg/mL for *S. littoralis*; 2.99 mg/mL for *A. ipsilon*); Shortening of larval and pupal stages and alteration of sex ratio in *S. littoralis*. - Effect linked mainly to eucalyptol and camphor, with contributions from α- and β-pinene.	[85]
	in vitro; in silico	EO	*Spodoptera frugiperda*	- Weak toxicity (LC_50_ = 2701.5 mg/L); synergy with cyantraniliprole (53.3%), antagonism with emamectin (36.7%). - Effect linked mainly to eucalyptol, camphor, and α- and β-pinene. - Docking confirmed binding of EO constituents, strongest for eucalyptol.	[111]
	in vitro	Acetone, hexane extracts	*Culex pipiens*	- Hexane extract: Strong larvicidal activity (LC_50_ = 0.08–0.15 ppm); acetone extract weaker (54–79 ppm). - Effect linked to: Non-polar compounds in the hexane extract	[112]
anti-hemolytic	in vitro	Aqueous extract	AAPH-induced hemolysis of rabbit erythrocytes	- Strong antihemolytic activity; increased half-time hemolysis by 479%. - Effect linked to polyphenols and flavonoids.	[74]
Cytotoxic	in vitro	Hexane, dichloromethane, methanol extracts	RD (embryonal rhabdomyosarcoma), BSR (hamster kidney adenocarcinoma), Vero (monkey kidney) cell lines	- Hexane and dichloromethane: Moderate cytotoxicity (IC_50_ = 115–300 µg/mL); methanol extract being inactive (>300 µg/mL). - Effect linked to Bioactive fatty acids (oleic acid, methyl linolenate) with possible synergistic effects.	[41]
Enzymes inhibition	in vitro	Crude extracts and fractions (dichloromethane, ethyl acetate, n-butanol)	α-amylase (anti-diabetic target), Butyrylcholinesterase (BuChE) (Alzheimer’s-related enzyme)	- α-amylase: Strong inhibition (antidiabetic potential) by crude extract (IC_50_ = 64.17 µg/mL), much stronger than acarbose (IC_50_ = 3650.93 µg/mL). - BuChE: Moderate inhibition (Alzheimer’s-related) by crude extract (IC_50_ = 83.55 µg/mL) and dichloromethane fraction (IC_50_ = 152.44 µg/mL). - Effect linked to polyphenols and tannins (α-amylase), alkaloids/terpenes (BuChE).	[75]

In addition, geographic and environmental factors have been shown to influence essential oil composition and antibacterial potential. A novel Tunisian ecotype was found to contain camphor (15.68%), 1,8-cineole (14.14%), and α-pinene (13.82%) as major constituents by Tofah et al. [43]. They evaluated its activity against *Escherichia coli* ATCC 8739 and *Staphylococcus aureus* ATCC 6583 using the agar well diffusion method, revealing notable efficacy (MIC = 0.625 μg/mL) with greater sensitivity in *E. coli* (inhibition diameter = 22.6 mm) compared to *S. aureus* (17.8 mm). *L. multifida* essential oil from Morocco (El Gharb region) was characterized by eucalyptol (28.11%), 2-bornanone (11.57%), endo-borneol (7.82%), and linalyl acetate (5.22%), exhibiting both antioxidant and antibacterial activities [113].

These collective results consistently position *L. multifida* essential oil as a potent antibacterial agent with varying degrees of efficacy across different bacterial species.

Beyond planktonic bacteria, *L. multifida* essential oil exhibits significant anti-biofilm properties. Alabdullatif et al. [110] documented dose-dependent biofilm inhibition against *Staphylococcus epidermidis*, achieving reductions of 1.319, 1.594, and 1.599 log CFU/mL at 10%, 20%, and 30% concentrations, respectively. Notably, the oil synergistically enhanced the efficacy of conventional disinfectants (chlorhexidine gluconate and isopropyl alcohol) in donor skin preparation protocols, highlighting its potential as an adjunct in clinical antisepsis.

*Lavandula multifida* essential oil exhibits broad-spectrum antifungal activity against both human pathogens and agricultural phytopathogens. Zuzarte et al. [34] demonstrated its potent efficacy against eighteen clinical isolates and reference strains from *Candida*, dermatophytes, *Aspergillus*, and *Cryptococcus* genera, with particularly strong activity against *Cryptococcus neoformans* and dermatophytes (MIC = 0.16 μL/mL). In agricultural applications, Sellam et al. [45] reported the complete growth inhibition of *Alternaria* sp. and *Rhizopus stolonifer* at 100 μg/mL, along with significant activity against *Penicillium expansum* and *Botrytis cinerea*—findings corroborated by Laghchimi et al. [84], who specifically documented its effectiveness against apple rot pathogens (*P. expansum*, *Alternaria* sp., and *R. stolonifer*). Recent work by Alves-Silva et al. [80] has expanded these findings to show notable antibiofilm properties against dermatophytes (*Epidermophyton floccosum*, *Trichophyton mentagrophytes*, *Microsporum gypseum*, *Trichophyton rubrum*) and *Candida albicans*, with carvacrol-rich oil demonstrating both inhibitory and disruptive effects on fungal biofilms.

It is evident from the above-mentioned studies that *L. multifida* exhibits a broad spectrum of activity against both Gram-positive and Gram-negative bacteria, as well as fungi pathogenic to humans and crops. Most investigations have focused on the essential oil, while comparatively fewer studies have examined organic extracts, which are potentially rich in phenolics, flavonoids, and terpenoids. The antibacterial activity of *L. multifida* is largely attributed to the major constituents of its essential oil, principally carvacrol, linalool, camphor, and durenol, which represent the dominant bioactive agents across different chemotypes, as evidenced by existing antibacterial studies. Among these, carvacrol, linalool, and camphor exert multiple, convergent mechanisms that collectively undermine bacterial survival. A principal mechanism involves the disruption of the cytoplasmic membrane, leading to altered permeability, ion leakage, dissipation of the membrane potential, and compromised structural integrity. This destabilization is closely linked to metabolic failure, including ATP depletion through the impairment of glycolysis, the tricarboxylic acid (TCA) cycle, and oxidative phosphorylation. In parallel, oxidative stress induction and respiratory interference further destabilize cellular homeostasis, amplifying the damage initiated at the membrane level. Additional mechanisms include biofilm inhibition, achieved by disrupting quorum-sensing pathways and weakening the extracellular matrix, as well as the modulation of enzymatic functions, such as β-lactamase inhibition, which restores antibiotic sensitivity [44,50,79,82,110,114]. Although several studies have documented the antibacterial activity of durenol [72,115,116,117], its underlying mechanisms of action remain insufficiently characterized. The antifungal activity of *L. multifida* is attributed primarily to carvacrol, which has been identified as its most active constituent in available antifungal investigations. Carvacrol exerts this activity through several complementary mechanisms. A central pathway involves the disruption of the fungal cell membrane, achieved either through direct interaction with membrane proteins or the inhibition of ergosterol biosynthesis; both mechanisms lead to increased permeability, the leakage of intracellular contents, and cell death. Complementing these effects, alterations in lipid metabolism and the modulation of antioxidant enzymes shift the cellular balance toward oxidative stress, further compromising fungal survival. In some cases, antifungal action also involves an initial phase of metabolic arrest preceding cell death. Furthermore, strong antibiofilm activity is achieved through a reduction in the extracellular matrix and capsule, thereby preventing biofilm establishment and promoting the disruption of mature biofilms [34,50,80,84].

It is important to note that the antimicrobial activity of *L. multifida* is not attributable to a single compound but rather reflects a synergistic interplay among constituents within the essential oil, a finding consistent with observations in other *Lavandula* species [31,118]. Studies on isolated constituents further confirm this synergy, as whole essential oils consistently exhibit stronger antimicrobial effects than their individual components [43,119]. This principle applies not only to interactions among phytochemicals within the essential oil but also to external combinations; for example, linalool enhances disinfectants such as chlorhexidine gluconate [110], and β-bisabolene potentiates antibiotics [79]. However, some studies have reported that certain major constituents of *Lavandula* essential oils can exhibit stronger activity than the whole oil itself [120,121].

Taken together, these findings position *L. multifida* as a potent antimicrobial agent, demonstrating consistent efficacy against both fungal and bacterial pathogens, with documented instances of competitive or superior performance compared to conventional antibiotics. These results not only validate the plant’s traditional uses but also highlight its potential for modern applications in clinical and agricultural settings, as well as its role as a synergistic enhancer of conventional disinfectants. Further investigations are needed to elucidate the contributions of poorly characterized non-volatile compounds and to achieve a more comprehensive understanding of their antimicrobial mechanisms.

### 6.2. Antioxidant Activity

Oxidative stress, resulting from the excessive production of free radicals (e.g., superoxide) and non-radical species (e.g., hydrogen peroxide) that overwhelm endogenous antioxidant defenses like glutathione (GSH) and superoxide dismutase (SOD), plays a critical role in the pathogenesis of cardiovascular, neurodegenerative, diabetic, and oncological diseases [122]. Extensive research has demonstrated the antioxidant potential of *L. multifida* essential oils and extracts. Mammeri et al. [75] conducted a comprehensive in vitro evaluation of Algerian *L. multifida* (Constantine and M’sila regions) crude extracts and their dichloromethane, ethyl acetate, and butanol fractions using multiple assays: 2,2-diphenyl-1-picrylhydrazyl (DPPH), Galvinoxyl, and ABTS radical scavenging; Cupric reducing antioxidant capacity (CUPRAC); Ferric Reducing Antioxidant Power (FRAP); phenanthroline reduction; and metal chelation. The ethyl acetate extract from Constantine exhibited notable DPPH scavenging activity (EC_50_ = 12.32 ± 0.82 μg/mL), though less potent than BHA (5.73 ± 0.41 μg/mL). M’sila’s ethyl acetate extract showed superior performance in ABTS (4.89 ± 0.20 μg/mL) and Galvinoxyl (9.60 ± 0.06 μg/mL) assays, along with exceptional reducing power in FRAP (1181.50 ± 8.64 µg AA equiv/mg extract), CUPRAC (5.8 ± 0.50 μg/mL), and phenanthroline (10.92 ± 3.31 µg/mL) tests. However, all extracts demonstrated limited metal chelation capacity (EC_50_ > 800 µg/mL), performing markedly worse than EDTA (8.80 ± 0.47 µg/mL), indicating that their antioxidant mechanism primarily involves free radical scavenging rather than metal ion sequestration.

Molina-Tijeras et al. [73] conducted a comprehensive evaluation of the antioxidant properties of Spanish *L. multifida* extracts, employing multiple standardized in vitro assays and extending the assessment to in vivo investigations in a high-fat-diet-induced obese mouse model. The in vitro assays demonstrated strong antioxidant potential, with a FRAP value of 2.576 mmol FeSO_4_ equivalents/g, a Trolox Equivalent Antioxidant Capacity (TEAC) of 1.30 mmol Trolox equivalents/g, and an oxygen radical absorbance capacity (ORAC) of 2.08 mmol Trolox equivalents/g. In the DPPH radical scavenging assay, the extract showed dose-dependent activity with an IC_50_ of 8.06 μg/mL, which was comparable to established antioxidants such as ascorbic acid (IC_50_ = 7.91 μg/mL), gallic acid (IC_50_ = 7.94 μg/mL), and epicatechin (IC_50_ = 6.43 μg/mL). Importantly, this antioxidant potential was further confirmed in vivo, where the administration of the extract to obese mice resulted in a significant reduction in hepatic lipid peroxidation, as reflected by lower TBARS (thiobarbituric acid reactive substances) levels.

Studies on Moroccan *L. multifida* have demonstrated significant but varied antioxidant potential across different plant extracts. Sellam et al. [45] reported that the essential oil exhibited moderate DPPH scavenging activity (IC_50_ = 16.83 ± 1.55 μg/mL), being approximately threefold less potent than BHT (IC_50_ = 7.73 ± 0.11 μg/mL). In the β-carotene/linoleic acid bleaching test at 100 μg/mL, the oil showed substantial lipid peroxidation inhibition (78.41 ± 1.22%), approaching but not matching BHT’s efficacy (86.2 ± 0.5%). Complementary research by Ramchoun et al. [74] on aqueous extracts revealed stronger radical scavenging capacity (DPPH IC_50_ = 2.6 ± 0.01 mg/mL) and notable ferric reducing power (FRAP value = 12.76 ± 0.48 mmol Trolox/g), suggesting that water-soluble antioxidants in *L. multifida* may contribute significantly to its overall antioxidant profile. These findings collectively indicate that *L. multifida* possesses multiple antioxidant mechanisms whose relative effectiveness depends on both the extraction method and specific antioxidant pathway being evaluated.

Comparative studies of Tunisian and Algerian *Lavandula* species have revealed significant differences in their antioxidant profiles. Messaoud et al. [81] evaluated *L. multifida*, *L. coronopifolia*, and *L. stoechas* using DPPH scavenging, FRAP, and metal chelation assays. Tunisian *L. multifida* demonstrated moderate essential oil activity (DPPH IC_50_ = 201.6 mg/mL; FRAP = 39.1 mmol/g) but substantially stronger methanol extract activity (DPPH IC_50_ = 19.3 mg/mL; FRAP = 377.8 mmol/g), with particularly effective metal chelation (essential oil IC_50_ = 7.9 mg/mL; methanol extract IC_50_ = 0.8 mg/mL), outperforming *L. stoechas* and competing with *L. coronopifolia*. Similarly, Dif et al. [46] found that Algerian *Lavandula* species exhibited varying antioxidant capacities in DPPH assays, with *L. dentata* (IC_50_ = 2.15 mg/mL) showing the strongest activity, followed by *L. multifida* (17.36 mg/mL) and *L. stoechas* (25.52 mg/mL). These comparative studies demonstrate that while *L. multifida* possesses notable antioxidant properties, its efficacy depends on extraction methods and shows geographic variation, with methanol extracts generally exhibiting superior activity to essential oils.

In summary, the evidence establishes *L. multifida* as a species with a robust antioxidant profile, consistently demonstrated in both in vitro and in vivo assays. This activity is largely attributed to its high phenolic content, particularly flavonoids like rutin, quercetin glucoside, and epicatechin gallate. In contrast, essential oils feature carvacrol as the major constituent, along with other terpenoids such as 1,8-cineole and caryophyllene oxide, as key antioxidant contributors. The species employs multiple mechanisms—including radical scavenging, reducing power, and the inhibition of lipid peroxidation—with a clear predominance of scavenging and reducing activities over metal chelation. Polyphenol-rich extracts consistently show stronger and more reproducible effects than essential oils, highlighting the primary role of phenolic compounds. Compared to other *Lavandula* species, its antioxidant performance is comparable to *L. coronopifolia*, stronger than *L. stoechas*, but weaker than *L. dentata*. Overall, *L. multifida* represents a valuable source of antioxidants, though its efficacy depends on extraction methods and may vary geographically.

### 6.3. Anti-Inflammatory Activity

Scientific studies validate the traditional use of *L. multifida* for inflammatory disorders. Sosa et al. [77] demonstrated that both ethanol and aqueous extracts significantly inhibit Croton oil-induced ear edema in mice. The ethanol extract was particularly potent, showing a 62% reduction in edema—only five times less effective than the reference drug—while the aqueous extract was weaker. This activity is primarily attributed to ethanol-soluble triterpenoids, including ursolic, oleanolic, and maslinic acid. These compounds act by suppressing histamine release, interfering with arachidonic acid metabolism, inhibiting elastase, modulating the complement cascade, and reducing nitric oxide production. The anti-inflammatory effect is supported by diterpenes like 15S,16-dihydroxyisopimar-8(9)-ene, which inhibit leukocyte activation, cyclooxygenase-2, pro-inflammatory cytokine release, and nitric oxide production. The monoterpene carvacrol further contributes by inhibiting cyclooxygenase and scavenging free radicals, thereby reducing oxidative stress-driven inflammation. Complementing these findings, Mammeri et al. [75] reported that an aqueous extract significantly reduced paw edema and pro-inflammatory mediator synthesis by up to 43% in vivo. This effect was attributed to flavonoid and phenolic constituents. Flavonoids inhibit cyclooxygenase pathways—reducing prostaglandin synthesis—and stabilize lysosomal membranes to prevent the release of pro-inflammatory enzymes. Phenolic compounds provide antioxidant effects that counteract oxidative stress and attenuate inflammation.

Overall, these results provide pharmacological support for the ethnomedicinal use of *L. multifida* in treating inflammation, with ethanol extracts generally exhibiting greater potency than aqueous preparations.

### 6.4. Insecticidal Activity

*Lavandula multifida* exhibits potent insecticidal properties against various agricultural pests. Awad et al. [85] demonstrated its essential oil’s toxicity against *Spodoptera littoralis* and *Agrotis ipsilon*, with LC_50_ values of 2.350 and 2.991 mg/mL after 96 h, respectively, indicating greater sensitivity in *S. littoralis*. This effect is primarily attributed to major monoterpenes (eucalyptol, camphor, α-pinene, and β-pinene), which disrupt ATP production, ion channels, and oxidative balance in insect cells. The oil also impairs key detoxification enzymes (α-esterase, glutathione S-transferase (GST), cytochrome P450), increasing insect vulnerability, though its precise molecular mechanism requires further investigation. Moustafa et al. [111] confirmed and expanded these findings against *Spodoptera frugiperda* larvae (LC_50_ = 2.70 mg/mL) and provided deeper mechanistic insight. The oil showed synergistic effects with cyantraniliprole but antagonism with emamectin benzoate. It reduced larval and pupal duration, suggesting the disruption of juvenile hormone signaling. Biochemical assays confirmed the modulation of detoxification enzymes, and molecular docking revealed the strong binding of eucalyptol to acetylcholinesterase (–6.77 kcal/mol) and cytochrome P450 (–7.84 kcal/mol), implicating interference with neurotransmission and detoxification. Furthermore, the larvicidal potential of *L. multifida* was confirmed against *Culex pipiens* mosquitoes, where a hexane extract demonstrated remarkable toxicity (LC_50_ = 0.15 ppm at 24 h), significantly outperforming an acetone extract (LC_50_ = 78.55 ppm) and disrupting larval development and adult reproduction [112].

Taken together, these findings highlight *L. multifida*’s broad-spectrum insecticidal activity, with particular efficacy against lepidopteran larvae and mosquitoes, supporting its potential as a natural pest control agent.

### 6.5. Other Pharmacological Activities

The antihemolytic potential of *L. multifida* was evaluated in vitro using a 2,2′-azobis (2-amidinopropane) dihydrochloride (AAPH)-induced rabbit erythrocyte model. Treatment with a polyphenol-rich aqueous extract increased the hemolysis half-time by 479%, indicating strong protection against oxidative damage. This effect is attributed primarily to flavonoids and other polyphenols, which neutralize free radicals and stabilize erythrocyte membranes by interacting with lipid bilayers [74].

Cytotoxicity screening by Aneb et al. [41] revealed the weak activity of hexane and dichloromethane extracts against RD (embryonal rhabdomyosarcoma), BSR (hamster kidney adenocarcinoma), and Vero cell lines, with IC_50_ values ranging from 115 to 300 μg/mL; the methanol extract was inactive. This activity may be due to bioactive fatty acids such as oleic acid and methyl linolenate, possibly enhanced by synergistic interactions with other phytoconstituents. Although the mechanisms are not fully understood, these compounds have been linked to anticancer effects via lipogenesis stimulation, the suppression of proliferation, and the induction of G1 arrest and apoptosis [123,124].

Beyond the described effects, Lavandula species have also exhibited anticancer properties. In this regard, in vitro studies by Fahmy et al. provided evidence that *Lavandula officinalis* essential oil negatively impacts cancer, exerting anti-proliferative effects in many different human cancer cell lines (hepatocellular carcinoma HepG2 cells, prostate cancer PC3 cells, A549 lung carcinoma cells, A431 skin cancer cells, HCT116 colon cancer cells and MCF7 breast cancer cells). The higher cytotoxic effect was observed on HepG2 and A549 cells. Indeed, the essential oil was able to promote 100% cell death in these cells at 100 µg/mL dose, reaching an IC_50_ equal to 67.8 and 12 µg/mL, respectively [125].

Such data were in accordance with previous studies demonstrating that the promising anti-neoplastic properties of lavender oils observed in Hodgkin’s lymphoma, human prostate cancer and A549, H1299, and C6 cancer cells were associated with the induction of apoptotic and necrotic cell death [126].

In line with these investigations, Zhao et al. also demonstrated that *Lavandula officinalis* essential oil, as well as its major constituent’s linalool and linalyl acetate, possess strong anticancer activities. Such effects were ascribed to the induction of G2/M phase arrest and the promotion of apoptotic cell death in human prostate cancer PC-3 cells. Notably, the essential oil and linalool also suppressed tumor growth in PC-3 derived xenograft tumor models by downregulating Ki67 and PCNA, two cell proliferation markers [127].

All together, these data suggest that *Lavandula* essential oil could have good anti-neoplastic potential that deserves to be better explored in the near future.

Nevertheless, current evidence remains limited, and further studies involving detailed phytochemical profiling and broader cytotoxic screening are needed to clarify the therapeutic potential of this species.

Enzyme inhibition assays revealed two key pharmacological activities: strong α-amylase inhibition (IC_50_ = 64 μg/mL), mainly due to tannins blocking catalytic sites, supported by flavonoids, and butyrylcholinesterase (BuChE) inhibition (IC_50_ = 83 μg/mL), linked to polyphenols, alkaloids, terpenes, and coumarins, suggesting potential relevance in Alzheimer’s disease management [75].

Ben Hadj Ahmed et al. [128] found no leishmanicidal activity in *L. multifida* essential oil against Leishmania major or *L. infantum* at concentrations up to 8 µg/mL. However, these results may be limited by chemotypic variation and a single assay type. Re-evaluation using chemically characterized oils and diverse biological models is recommended.

Beyond pharmacology, *L. multifida* essential oil has industrial applications: it acts as a corrosion inhibitor for C38 steel in acidic media, showing 72.2% inhibition at 2 g/L via spontaneous Langmuir adsorption [83].

The biological activities of *L. multifida* are largely ascribed to its diverse phytochemical composition, including mono- and sesquiterpenes, polyphenols, flavonoids, triterpenoids, and fatty acids. These classes are associated with broad properties such as antitumor, antimutagenic, anti-angiogenic, analgesic, anti-elastase, cardioprotective, neuroprotective, antidiabetic, hepatoprotective, antiviral, insecticidal, and antiparasitic effects [129,130,131,132,133,134,135,136]. Although lavender species are generally low in toxicity—with rare cases of allergic contact dermatitis reported [60]—carvacrol, a key component, exhibits selective antimicrobial activity without harming human cells at bioactive concentrations, supporting its safety for potential therapeutic use. Overall, *L. multifida* represents a promising, though underexplored, source of bioactive compounds deserving further study.

## 7. Conclusions

This systematic review consolidates the extensive phytochemical and pharmacological research on *Lavandula multifida*, a species of significant ethnobotanical importance in the Mediterranean region. *L. multifida* possesses a distinctive and variable phytochemical profile, dominated by phenolic monoterpenes such as carvacrol. This composition aligns its chemistry and bioactivity more closely with genera like *Origanum* and *Thymus* than with typical lavender species. The essential oil also contains significant levels of β-bisabolene, 1,8-cineole, linalool, and camphor, though their proportions vary due to geographic, environmental, and methodological factors. Equally important is the non-volatile fraction, which is rich in flavonoids, triterpenoids, and phenolic acids that contribute strongly to its antioxidant activity and broaden its pharmacological potential.

The most robustly demonstrated property of *L. multifida* is its broad-spectrum antimicrobial activity, which stems from its complex phytochemistry. Bioactive compounds like carvacrol and linalool act synergistically to exert potent effects against diverse pathogens, including multi-drug-resistant bacteria and fungi. Their mechanisms include membrane disruption, metabolic interference, and the inhibition of biofilm formation.

Beyond antimicrobial applications, the plant exhibits significant antioxidant and anti-inflammatory properties, primarily due to its non-volatile constituents. The high phenolic content in organic extracts confers potent radical-scavenging and reducing power, often comparable to synthetic antioxidants. Additionally, triterpenoids, flavonoids, and carvacrol contribute to anti-inflammatory effects by modulating key pathways such as cyclooxygenase activity, cytokine release, and nitric oxide production, thereby reducing edema and validating traditional uses.

Despite these promising properties, research on *L. multifida* remains excessively focused on its essential oil, leaving the pharmacology of its non-volatile compounds underexplored. Future studies should prioritize mechanistic investigations of isolated bioactive molecules, rigorous preclinical in vivo and toxicological assessments, and eventually clinical trials. The standardization of extracts to account for chemotypic variation and the validation of traditional applications—such as for diabetes or cancer—are also essential.

In conclusion, *L. multifida* represents a phytochemically unique and pharmacologically promising species, supported by scientific evidence of its antimicrobial, antioxidant, and anti-inflammatory effects. Its chemical richness and efficacy against resistant pathogens position it as a compelling candidate for developing standardized herbal products, adjuvant therapies, and natural agrochemicals. However, realizing its full potential requires an integrated research approach that emphasizes its non-volatile compounds, detailed mechanistic studies, and thorough preclinical validation.

## Figures and Tables

**Table 1:** Traditional uses of *L. multifida* in the Mediterranean area.

Countries	Local Names	Plant Parts Used	Traditional Uses	Preparation Form	Ref.
Algeria	Kammoun el djmel, Tay djebal, Zeriga, Khzama	Aerial parts, leaf	Influenza, hypotensive, Sedative, stomachic, antispasmodic, astringent and cancer	Infusion, Cataplasm for head, Ocular drip	[38,39,67]
Spain	Cantueso, Cantagueso, Cantigueso	Aerial parts	Digestive problem and fatigue	Infusion	[66]
Morocco	Kohyla, Kohayla, Hlihla	Aerial parts	Broncho-pulmonary affections, Rheumatism, chill, digestive system, cough, cold, hepatitis, icterus	Decoction, Infusion, Pow	[41,42,68]
Portugal	alfazema-de-folhas-recortadas, Alfazema de folha recortada	Flowering aerial parts, flowers and stems.	bronchitis, asthma, and cough; digestion and bile stimulation; nervousness and dizziness; soporific; carminative; tonic; and hair stimulation	* NI	[34,39]
Tunisia	Soltan oud, Kammoun	Leaves, Flowers	Hypotensive, emmenagogue, antidiabetic, and hair perfume	Decoction, Maceration	[65,69]

* NI: Not Indicated.

**Table 2:** The phytochemical compounds isolated from *L. multifida*.

Country of Origin	Plant Part	Used Extract	Analytical Method	Chemical Classes	Identified Compounds	Ref.
Algeria	Aerial parts	Methanol extract	HPLC–DAD	Phenolic acids	Protocatechuic acid, caffeic acid	[76]
				Flavonoids	Apigenin, rutin, catechin	
				Coumarin	Coumarin	
		Petroleum Ether extract	GC–FID	Fatty acids	Palmitic acid, stearic acid, myristic acid, caprylic acid, dodecanoic acid, pentadecanoic acid, 14-methyl-hexadecanoic acid, margaric acid, nonadecanoic acid, eicosanoic acid, oleic acid, linoleic acid, palmitoleic acid.	
Morocco	Aerial parts	Ethanol extract	HPLC, TLC, RP-HPLC, NMR	Monoterpenes	Carvacrol, Carvacrol-3-glucoside	[77]
				Diterpenes	15S,16-dihydroxy-7-oxopimar-8(9)-ene, 15,16,17-trihydroxy-7-oxopimar-8(9)-ene, 15,16-dihydroxy-7,11-dioxopimar-8(9)-ene, 15,16,17-trihydroxypimar-8(9)-ene, 15S,16-dihydroxyisopimar-8(9)-ene	
				Triterpenes	Maslinic acid, Oleanolic acid, Ursolic acid, 3β,19α,23-trihydroxy-urs-12-en-28-oic acid	
Spain	Aerial parts	Hydrometh- anolic extract	UHPLC-MS	Phenolic acids	Mucic acid lactone gallate, dihydroferulic acid glucuronide, chicoric acid, amurensin	[73]
				Flavonoids	Hesperetin, quercetin glucoside, quercetin glucuronide, luteolin-7-O-glucoside, kaempferol acetyl-glucopyranoside, isorhamnetin-3-O-glucuronide, isoscutellarin-8-O-glucuronide, rutin, (epi)catechin digallate	
				Triterpenes	Maslinic acid (and isomers), madecassic acid (and isomers), asiatic acid (and isomers), quillaic acid (and isomers), glycyrrhetinic acid, camellenodiol	
				Saponins	Licoricesaponin J2 (or isomer), bryoamaride (or isomer), yunganoside G2 (or isomer), hovenidulcigenin B (or isomer)	
				Fatty acids	Fatty acid (C_18_H_32_O_3_), fatty acid (C_18_H_30_O_3_), fatty acid (C_18_H_30_O_2_), fatty acid (unspecified formulas)	
				Sterols/Steroidal derivatives	Stigmastene dione, hydroxyecdysone monoacetonide	
				Other metabolites	Citreaglycon A, malic acid,	
Italy	Fresh leaves	Methanolic extract	RP-DAD-HPLC	Monoterpene	Carvacrol	[42]
				Flavonoids (flavones and derivatives)	Vitexin (apigenin-8-C-glucoside), hypolaetin-7-O-glucoside, scutellarein-7-O-glucoside, luteolin-7-O-glucoside, isoscutellarein-7-O-glucoside, apigenin-7-O-glucoside, chrysoeriol-7-O-glucoside, isoscutellarein-8-O-glucoside, apigenin	

## Data Availability

Not applicable.

## References

[1] Jindal A., Seth C.S. (2022). Medicinal plants: The rising strategy for synthesis of modern medicine. Int. J. Plant Environ..

[2] Dhayalan M., Anitha Jegadeeshwari L., Nagendra Gandhi N. (2015). Biological activity sources from traditionally used tribe and herbal plants material. Asian J. Pharm. Clin. Res..

[3] Ravichandran S., Bhargavi K.M., Rai A., Pandey T., Rajput J., Sri R.M.M. (2023). Medicinal plants for curing human diseases. Insight-Chin. Med..

[4] Sadiq I.Z., Abubakar F.S., Ibrahim B., Usman M.A., Kudan Z.B. (2019). Medicinal plants for management and alternative therapy of common ailments in Dutsin-Ma (Katsina State) in Nigeria. Herba Pol..

[5] Jamshidi-Kia F., Lorigooini Z., Amini-Khoei H. (2018). Medicinal plants: Past history and future perspective. J. Herbmed Pharmacol..

[6] Ogunrinola O.O., Kanmodi R.I., Ogunrinola O.A. (2022). Medicinal plants as immune booster in the palliative management of viral diseases: A perspective on coronavirus. Food Front..

[7] González-Tejero M.R., Casares-Porcel M., Sánchez-Rojas C.P., Ramiro-Gutiérrez J.M., Molero-Mesa J., Pieroni A., Giusti M.E., Censorii E., de Pasquale C., Della A. (2008). Medicinal plants in the Mediterranean area: Synthesis of the results of the project Rubia. J. Ethnopharmacol..

[8] Miara M.D., Bendif H., Ait Hammou M., Teixidor-Toneu I. (2018). Ethnobotanical survey of medicinal plants used by nomadic peoples in the Algerian steppe. J. Ethnopharmacol..

[9] Aboukhalaf A., Tbatou M., Kalili A., Naciri K., Moujabbir S., Sahel K., Rocha J.M., Belahsen R. (2022). Traditional knowledge and use of wild edible plants in Sidi Bennour region (Central Morocco). Ethnobot. Res. Appl..

[10] Savvides A.M., Stavridou C., Ioannidou S., Zoumides C., Stylianou A. (2023). An ethnobotanical investigation into the traditional uses of mediterranean medicinal and aromatic plants: The case of troodos mountains in Cyprus. Plants.

[11] Marín J., Garnatje T., Vallès J. (2023). Traditional knowledge 10 min far from Barcelona: Ethnobotanical study in the Llobregat river delta (Catalonia, NE Iberian Peninsula), a heavily anthropized agricultural area. J. Ethnobiol. Ethnomed..

[12] Patti M., Musarella C.M., Spampinato G. (2025). Ethnobotanical knowledge in Calabria (southern Italy): A summary review. Heliyon.

[13] Fakir H., Korkmaz M., Güller B. (2009). Medicinal plant diversity of western Mediterranean region in Turkey. J. Appl. Biol. Sci..

[14] Nawash O.S., Al-Assaf A., El-Oqlah A., Omari M. (2014). Floristic features, distribution, and ethnobotany of plants gathered and used by local people from the Mediterranean forest in northern Jordan. Ethnobot. Res. Appl..

[15] de Cortes Sánchez-Mata M., Tardío J. (2016). Mediterranean Wild Edible Plants: Ethnobotany and Food Composition Tables.

[16] Batiha G.E.-S., Teibo J.O., Wasef L., Shaheen H.M., Akomolafe A.P., Teibo T.K.A., Al-Kuraishy H.M., Al-Garbeeb A.I., Alexiou A., Papadakis M. (2023). A Review of the bioactive components and pharmacological properties of *Lavandula* species. Naunyn-Schmiedeberg’s Arch. Pharmacol..

[17] Bayındır D., Uysal G., Erbaş S., Devran Z. (2023). The response of lavender and lavandin cultivars to *Meloidogyne incognita* and *Meloidogyne arenaria*. J. Plant Dis. Prot..

[18] Sun M., Zhu L., Zhang Y., Liu N., Zhang J., Li H., Bai H., Shi L. (2023). Creation of new germplasm resources, development of SSR markers, and screening of monoterpene synthases in thyme. BMC Plant Biol..

[19] Ricardo-Rodrigues S., Rouxinol M.I., Agulheiro-Santos A.C., Potes M.E., Laranjo M., Elias M. (2024). The antioxidant and antibacterial potential of thyme and clove essential oils for meat preservation—An overview. Appl. Biosci..

[20] Boulares M., Bezzezi A., Arfaoui M., Boulares A., Ghrab M., Ben Moussa O., Hassouna M., Boudiche S. (2022). Improvement of Tunisian ‘Chemlali’ extra virgin olive oil stability with rosemary and laurel herbs and essential oils. Riv. Ital. Sostanze Grasse.

[21] Barak T.H., Bölükbaş E., Bardakci H. (2023). Evaluation of marketed rosemary essential oils (*Rosmarinus officinalis* L.) in terms of European Pharmacopoeia 10.0 criteria. Turk. J. Pharm. Sci..

[22] Silva B.N., Cadavez V., Caleja C., Pereira E., Calhelha R.C., Añibarro-Ortega M., Finimundy T. (2023). Phytochemical composition and bioactive potential of *Melissa officinalis* L., *Salvia officinalis* L., and *Mentha spicata* L. extracts. Foods.

[23] Napoli E., Kim M., Sowndhararajan K., Ruberto G., Kim S. (2023). The effect of exposure to *Mentha suaveolens* Ehrh. essential oil on the electroencephalographic activity according to gender difference. J. Essent. Oil Res..

[24] Qanash H., Bazaid A.S., Aldarhami A., Alharbi B., Almashjary M.N., Hazzazi M.S., Felemban H.R., Abdelghany T.M. (2023). Phytochemical characterization and efficacy of *Artemisia judaica* extract loaded chitosan nanoparticles as inhibitors of cancer proliferation and microbial growth. Polymers.

[25] Lantzouraki D.Z., Amerikanou C., Karavoltsos S., Kafourou V., Sakellari A., Tagkouli D., Zoumpoulakis P., Makris D.P., Kalogeropoulos N., Kaliora A.C. (2023). *Artemisia arborescens* and *Artemisia inculta* from Crete; secondary metabolites, trace metals and in vitro antioxidant activities. Life.

[26] Barut M., Tansi L.S. (2024). Elucidating the flower and seed yield and phytochemical variability of marigold (*Calendula officinalis* L.) in response to winter sowing at different harvest intervals and dates. S. Afr. J. Bot..

[27] Barut M., Tansi L.S., Karaman Ş. (2023). Unveiling the phytochemical variability of fatty acids in world marigold (*Calendula officinalis* L.) germplasm affected by genotype. Int. J. Agric. Environ. Food Sci..

[28] Guitton Y., Nicolè F., Jullien F., Caissard J.-C., Saint-Marcoux D., Legendre L., Pasquier B., Moja S. (2018). A comparative study of terpene composition in different clades of the genus *Lavandula*. Bot. Lett..

[29] Salehi B., Mnayer D., Özçelik B., Altin G., Kasapoğlu K.N., Daskaya-Dikmen C., Sharifi-Rad M. (2018). Plants of the genus *Lavandula*: From farm to pharmacy. Nat. Prod. Commun..

[30] Aprotosoaie A.C., Gille E., Trifan A., Luca V.S., Miron A. (2017). Essential oils of *Lavandula* genus: A systematic review of their chemistry. Phytochem. Rev..

[31] Ez zoubi Y., Bousta D., Farah A. (2020). A phytopharmacological review of a Mediterranean plant: *Lavandula stoechas* L.. Clin. Phytosci..

[32] Héral B., Stierlin É., Fernandez X., Michel T. (2021). Phytochemicals from the genus *Lavandula*: A review. Phytochem. Rev..

[33] Vairinhos J., Miguel M.G. (2020). Essential oils of spontaneous species of the genus *Lavandula* from Portugal: A brief review. Z. Naturforsch. C.

[34] Zuzarte M., Vale-Silva L., Gonçalves M.J., Cavaleiro C., Vaz S., Canhoto J., Pinto E., Salgueiro L. (2012). Antifungal activity of phenolic-rich *Lavandula multifida* L. essential oil. Eur. J. Clin. Microbiol. Infect. Dis..

[35] Benabdelkader T., Zitouni A., Guitton Y., Jullien F., Maitre D., Casabianca H., Legendre L., Kameli A. (2011). Essential oils from wild populations of Algerian *Lavandula stoechas* L.: Composition, chemical variability, and in vitro biological properties. Chem. Biodivers..

[36] Benbelaid D.E.A.F., Bendahou M., Khadir A., Abdoune M.A., Bellahsene C., Zenati F., Bouali W. (2012). Antimicrobial activity of essential oil of *Lavandula multifida* L.. J. Microbiol. Biotechnol. Res..

[37] Mechaala S., Bouatrous Y., Adouane S. (2022). Traditional knowledge and diversity of wild medicinal plants in El Kantara’s area (Algerian Sahara gate): An ethnobotany survey. Acta Ecol. Sin..

[38] Hendel N., Larous L., Sari M., Boudjelal A., Sarri D. (2012). Place of labiates in folk medicine of the area of M’sila (Algeria). Glob. J. Res. Med. Plants Indig. Med..

[39] Neves J.M., Matos C., Moutinho C., Queiroz G., Gomes L.R. (2009). Ethnopharmacological notes about ancient uses of medicinal plants in Trás-os-Montes (northern of Portugal). J. Ethnopharmacol..

[40] Bachiri L., Labazi N., Daoudi A., Ibijbijien J., Nassiri L., Echchegadda G., Mokhtari F. (2015). Ethnobotanical study of some spontaneous moroccan lavenders. Int. J. Biol. Chem. Sci..

[41] Aneb M., Talbaoui A., Bouyahya A., El Boury H., Amzazi S., Benjouad A., Dakka N., Bakri Y. (2016). In vitro cytotoxic effects and antibacterial activity of moroccan medicinal plants *Aristolochia longa* and *Lavandula multifida*. Eur. J. Med. Plants.

[42] Panuccio M.R., Fazio A., Papalia T., Barreca D. (2016). Antioxidant properties and flavonoid profile in leaves of calabrian *Lavandula multifida* L., an autochthon plant of mediterranean southern regions. Chem. Biodivers..

[43] Tofah M.L., Mseddi K., Al-Abbasi O.K., Ben Yazid A., Khechine A., Gdoura R., Khannous L. (2023). A new lavender (*Lavandula multifida* L.) ecotype from arid Tunisia, with differential essential oil composition and higher antimicrobial potential. Life.

[44] Elmakaoui A., Bourais I., Oubihi A., Nassif A., Bezhinar T., Shariati M.A., Blinov A.V., Hleba L., El Hajjaji S. (2022). Chemical composition and antibacterial activity of essential oil of *Lavandula multifida*. J. Microbiol. Biotechnol. Food Sci..

[45] Sellam K., Ramchoun M., Alem C., ElRhaffari L. (2013). Biological investigations of antioxidant-antimicrobial properties and chemical composition of essential oil from *Lavandula multifida*. Oxid. Antioxid. Med. Sci..

[46] Dif M.M., Benyahia M., Toumi Benali F., Rahmani M., Bouazza S. (2017). Teneur en composés phénoliques et activité antioxydantes de trois espèces algérienne de *Lavandula*. Phytotherapie.

[47] Gross N., Börger L., Soriano-Morales S.I., Le Bagousse-Pinguet Y., Quero J.L., García-Gómez M., Valencia-Gomez E., Maestre F.T. (2013). Uncovering Multiscale Effects of Aridity and Biotic Interactions on the Functional Structure of Mediterranean Shrublands. J. Ecol..

[48] García-Caparrós P., Llanderal A., Pestana M., Correia P.J., Lao M.T. (2017). *Lavandula multifida* response to salinity: Growth, nutrient uptake, and physiological changes. J. Plant Nutr. Soil Sci..

[49] Nuutinen T. (2018). Medicinal Properties of Terpenes Found in *Cannabis sativa* and *Humulus lupulus*. Eur. J. Med. Chem..

[50] Mączka W., Twardawska M., Grabarczyk M., Wińska K. (2023). Carvacrol—A Natural Phenolic Compound with Antimicrobial Properties. Antibiotics.

[51] Hoch C.C., Petry J., Griesbaum L., Weiser T., Werner K., Ploch M., Verschoor A., Multhoff G., Dezfouli A.B., Wollenberg B. (2023). 1,8-Cineole (Eucalyptol): A Versatile Phytochemical with Therapeutic Applications across Multiple Diseases. Biomed. Pharmacother..

[52] Zuzarte M., Dinis A.M., Cavaleiro C., Canhoto J., Salgueiro L. (2008). Trichomes morphology and essential oils characterization of field-growing and in vitro propagated plants of *Lavandula pedunculata*. Microsc. Microanal..

[53] Znini M., Laghchimi A., Paolini J., Costa J., Majidi L. (2019). Characterization of *Lavandula multifida* volatile composition from Morocco by headspace solid-phase microextraction (HS-SPME) and hydrodistillation coupled to GC–MS. Arab. J. Med. Aromat. Plants.

[54] Lacaze R. (1965). La culture de la lavande dans le Quercy. Rev. Géogr. Pyrénées Sud-Ouest.

[55] Msaada K., Salem N., Tammar S., Hammami M., Saharkhiz M.J., Debiche N., Limam F., Marzouk B. (2012). Essential oil composition of *Lavandula dentata*, *L. stoechas* and *L. multifida* cultivated in Tunisia. J. Essent. Oil-Bear. Plants.

[56] Plants of the World Online (POWO) (2025). Lavandula multifida L.. https://powo.science.kew.org/taxon/449064-1.

[57] World Flora Online (WFO) (2025). Lavandula multifida L.. http://www.worldfloraonline.org/taxon/wfo-0000224197.

[58] Fazio A., Cerezuela R., Panuccio M.R., Cuesta A., Esteban M.Á. (2017). In vitro effects of Italian *Lavandula multifida* L. leaf extracts on gilthead seabream (*Sparus aurata*) leucocytes and SAF-1 cells. Fish Shellfish Immunol..

[59] Lis-Balchin M. (2002). Lavender: The Genus Lavandula.

[60] Upson T.M., Jury S.L. (2002). A revision of native Moroccan species of *Lavandula* L. section *Pterostoechas* Ging. (Lamiaceae). Taxon.

[61] Panuccio M.R., Fazio A., Musarella C.M., Mendoza-Fernández A.J., Mota J.F., Spampinato G. (2018). Seed germination and antioxidant pattern in *Lavandula multifida* (Lamiaceae): A comparison between core and peripheral populations. Plant Biosyst..

[62] Chograni H., Zaouali Y., Rajeb C., Boussaid M. (2010). Essential oil variation among natural populations of *Lavandula multifida* L. (Lamiaceae). Chem. Biodivers..

[63] Ghadiri M.K., Gorji A. (2002). Lavender for medicine: A brief review of clinical effects. Avicenna.

[64] El Rhaffari L., Ismaïli-Alaoui M., Belkamel J., Jeannot V. (2007). Chemical composition and antibacterial properties of the essential oil of *Lavandula multifida* L.. Int. J. Essent. Oil Ther..

[65] Karous O., Jilani I.B.H., Ghrabi-Gammar Z. (2021). Ethnobotanical study on plant used by semi-nomad descendants’ community in Ouled Dabbeb—Southern Tunisia. Plants.

[66] Martínez-Lirola M.J., González-Tejero M.R., Molero-Mesa J. (1996). Ethnobotanical resources in the province of Almería, Spain: Campos de Nijar. Econ. Bot..

[67] Bourlière F., Quezel P., Santa S. (1963). Nouvelle Flore de l’Algérie et de Ses Régions Désertiques Méridionales. Tome II.

[68] El-Hilaly J., Hmammouchi M., Lyoussi B. (2003). Ethnobotanical Studies and Economic Evaluation of Medicinal Plants in Taounate Province (Northern Morocco). J. Ethnopharmacol..

[69] Fatma G., Sami B.H.A., Ahmed L. (2017). Investigation of extracts from Tunisian ethnomedicinal plants as antioxidants, cytotoxins, and antimicrobials. Biomed. Environ. Sci..

[70] Koblovská R., Macková Z., Vítková M., Kokoška L., Klejdus B., Lapčík O. (2008). Isoflavones in the rutaceae family: Twenty selected representatives of the genera *Citrus*, *Fortunella*, *Poncirus*, *Ruta*, and *Severinia*. Phytochem. Anal..

[71] Lee C.J., Chen L.G., Chang T.L., Ke W.M., Lo Y.F., Wang C.C. (2011). The correlation between skin-care effects and phytochemical contents in lamiaceae plants. Food Chem..

[72] Soro N.K., Majdouli K., Moutaouakil K., Elhilali F., Khabbal Y., Bentayeb A., Zaïr T. (2014). Chemical composition and antibacterial power of *Lavandula multifida* L. essential oil against multiresistant strains of *Escherichia coli*, *Pseudomonas aeruginosa*, and *Klebsiella pneumoniae* isolated in hospital. Int. J. Innov. Sci. Res..

[73] Molina-Tijeras J.A., Ruiz-Malagón A.J., Hidalgo-García L., Diez-Echave P., Rodríguez-Sojo M.J., Cádiz-Gurrea M.L., Segura-Carretero A. (2023). The antioxidant properties of *Lavandula multifida* extract contribute to its beneficial effects in High-Fat Diet-Induced obesity in mice. Antioxidants.

[74] Ramchoun M., Harnafi H., Alem C., Benlyas M., Elrhaffari L., Amrani S. (2009). Study on antioxidant and hypolipidemic effects of polyphenol-rich extracts from *Thymus vulgaris* and *Lavandula multifida*. Pharmacogn. Res..

[75] Mammeri A., Bendif H., Bensouici C., Benslama A., Rebas K., Bouasla A., Rebaia I., Souilah N., Miara M.D. (2022). Total phenolic contents, in vitro antioxidant activity, enzyme inhibition and anti-inflammatory effect of the selective extracts from the algerian *Lavandula multifida*. Acta Pharm. Sci..

[76] Soudani L., Nabi F., Bendif H., Dilaycan Ç., Bouriah N., Öztürk M. (2025). In-Depth Chemical profile by GC–MS, ICP–OES and HPLC–DAD, and In Vitro antioxidant properties of *Lavandula multifida*. Food Anal. Methods.

[77] Sosa S., Altinier G., Politi M., Braca A., Morelli I., Della Loggia R. (2005). Extracts and constituents of *Lavandula multifida* with topical anti-inflammatory activity. Phytomedicine.

[78] Saadi A., Brada M., Kouidri M., Dekkiche H., Attar F. (2016). Chemical composition and content of essential oil of *Lavandula multifida* from Algeria. Chem. Nat. Compd..

[79] Khadir A., Bendahou M., Benbelaid F., Abdoune M.A., Bellahcene C., Zenati F., Muselli A., Paolini J., Costa J. (2016). Chemical composition and Anti-MRSA activity of essential oil and ethanol extract of *Lavandula multifida* L. from Algeria. J. Essent. Oil-Bear. Plants.

[80] Alves-Silva J., Zuzarte M., Cavaleiro C., Salgueiro L. (2023). Antibiofilm effect of *Lavandula multifida* essential oil: A new approach for chronic infections. Pharmaceutics.

[81] Messaoud C., Chograni H., Boussaid M. (2013). Chemical composition and antioxidant activities of essential oils and methanol extracts of three wild *Lavandula* L. Species. Nat. Prod. Res..

[82] Douhri B., Douhri H., Farah A., Idaomar M., Senhaji N.S., Abrini J. (2014). Phytochemical analysis and antibacterial activity of essential oil of *Lavandula multifida* L.. Int. J. Innov. Sci. Res..

[83] Znini M., Paolini J., Majidi L., Desjobert J.-M., Costa J., Lahhit N., Bouyanzer A. (2012). Evaluation of the inhibitive effect of *Lavandula multifida* L. essential oil on the corrosion behavior of C38 steel in 0.5 M H_2_SO_4_ medium. Res. Chem. Intermed..

[84] PubChem PubChem Database. National Center for Biotechnology Information. https://pubchem.ncbi.nlm.nih.gov/.

[85] Laghchimi A., Znini M., Majidi L., Renucci F., El Harrak A., Costa J. (2014). Composition chimique et effet des phases liquide et vapeur de l’huile essentielle de *Lavandula multifida* sur la croissance mycélienne des moisissures responsables de la pourriture de la pomme. J. Mater. Environ. Sci..

[86] Awad M., Moustafa M.A.M., Alfuhaid N.A., Amer A., Ahmed F.S. (2024). Toxicological, biological, and biochemical impacts of the egyptian lavender (*Lavandula multifida*) essential oil on two lepidopteran pests. J. Plant Prot. Res..

[87] Wells R., Truong F., Adal A.M., Sarker L.S., Mahmoud S.S. (2018). *Lavandula* essential oils: A current review of applications in medicinal, food, and cosmetic industries of lavender. Nat. Prod. Commun..

[88] Zenão S., Aires A., Dias C., Saavedra M.J., Fernandes C. (2017). Antibacterial potential of *Urtica dioica* and *Lavandula angustifolia* extracts against methicillin-resistant *Staphylococcus aureus* isolated from diabetic foot ulcers. J. Herb. Med..

[89] Bouyahya A., Et-Touys A., Abrini J., Talbaoui A., Fellah H., Bakri Y., Dakka N. (2017). *Lavandula stoechas* essential oil from Morocco as novel source of antileishmanial, antibacterial, and antioxidant activities. Biocatal. Agric. Biotechnol..

[90] Pombal S., Rodrigues C.F., Araújo J.P., Rocha P.M., Rodilla J.M., Diez D., Granja Á.P., Gomes A.C., Silva L.A. (2016). Antibacterial and antioxidant activity of Portuguese *Lavandula luisieri* (Rozeira) Rivas-Martinez and its relation with their chemical composition. Springerplus.

[91] Zuzarte M., Gonçalves M.J., Cruz M.T., Cavaleiro C., Canhoto J., Vaz S., Pinto E., Salgueiro L. (2012). *Lavandula luisieri* essential oil as a source of antifungal drugs. Food Chem..

[92] Carrasco Ruiz A., Tomas V., Tudela J., Miguel M.G. (2016). Comparative study of GC-MS characterization, antioxidant activity, and hyaluronidase inhibition of different species of *Lavandula* and *Thymus* essential oils. Flavour Fragr. J..

[93] Nikolova G., Karamalakova Y., Kovacheva N., Stanev S., Zheleva A., Gadjeva V. (2016). Protective effect of two essential oils isolated from *Rosa damascena* Mill. and *Lavandula angustifolia* Mill., and two classic antioxidants against L-dopa oxidative toxicity induced in healthy mice. Regul. Toxicol. Pharmacol..

[94] Georgiev Y.N., Paulsen B.S., Kiyohara H., Ciz M., Ognyanov M.H., Vasicek O., Rise F. (2017). The common lavender (*Lavandula angustifolia* Mill.) pectic polysaccharides modulate phagocytic leukocytes and intestinal Peyer’s Patch Cells. Carbohydr. Polym..

[95] Husseini Y., Sahraei H., Meftahi G.H., Dargahian M., Mohammadi A., Hatef B., Zardooz H. (2016). Analgesic and anti-inflammatory activities of hydro-alcoholic extract of *Lavandula officinalis* in mice: Possible involvement of the cyclooxygenase Type 1 and 2 Enzymes. Rev. Bras. Farmacogn..

[96] Panahi Y., Akhavan A., Sahebkar A., Hosseini S.M., Taghizadeh M., Akbari H., Sharif M.R., Imani S. (2014). Investigation of the effectiveness of *Syzygium aromaticum*, *Lavandula angustifolia*, and *Geranium robertianum* essential oils in the treatment of acute external otitis: A comparative trial with ciprofloxacin. J. Microbiol. Immunol. Infect..

[97] Al Sufyani N.M., Hussien N.A., Hawsawi Y.M. (2019). Characterization and anticancer potential of silver nanoparticles biosynthesized from *Olea chrysophylla* and *Lavandula dentata* leaf extracts on HCT116 colon cancer cells. J. Nanomater..

[98] Miastkowska M., Kantyka T., Bielecka E., Kałucka U., Kamińska M., Kucharska M., Kilanowicz A., Cudzik D., Cudzik K. (2021). Enhanced biological activity of a novel preparation of *Lavandula angustifolia* essential oil. Molecules.

[99] Rabiei Z., Rafieian-Kopaei M., Mokhtari S., Alibabaei Z., Shahrani M. (2014). The Effect of pretreatment with different doses of *Lavandula officinalis* ethanolic extract on memory, learning, and nociception. Biomed. Aging Pathol..

[100] Costa P., Sarmento B., Gonçalves S., Romano A. (2013). Protective effects of *Lavandula viridis* L’Hér extracts and rosmarinic acid against H_2_O_2_-induced oxidative damage in A172 Human astrocyte cell line. Ind. Crops Prod..

[101] Videira R., Castanheira P., Grãos M., Salgueiro L., Faro C., Cavaleiro C. (2013). A Necrodane monoterpenoid from *Lavandula luisieri* essential oil as a cell-permeable inhibitor of BACE-1, the β-Secretase in Alzheimer’s disease. Flavour Fragr. J..

[102] Seyyed-Rasooli A., Salehi F., Mohammadpoorasl A., Goljaryan S., Seyyedi Z., Thomson B. (2016). Comparing the effects of aromatherapy massage and inhalation aromatherapy on anxiety and pain in burn patients: A single-blind randomized clinical trial. Burns.

[103] Bagheri-Nesami M., Espahbodi F., Nikkhah A., Shorofi S.A., Charati J.Y. (2014). The effects of lavender aromatherapy on pain following needle insertion into a fistula in hemodialysis patients. Complement. Ther. Clin. Pract..

[104] Soltani R., Soheilipour S., Hajhashemi V., Asghari G., Bagheri M., Molavi M. (2013). Evaluation of the effect of aromatherapy with lavender essential oil on post-tonsillectomy pain in pediatric patients: A randomized controlled trial. Int. J. Pediatr. Otorhinolaryngol..

[105] İşlek Z., Şahin F. (2023). In Vitro Antileishmanial Activity of *Lavandula angustifolia* Essential Oil on *Leishmania infantum* Parasites. Experimed.

[106] Yao N., He J.K., Pan M., Hou Z.F., Xu J.J., Yang Y., Tao J.P., Huang S.Y. (2021). In Vitro Evaluation of *Lavandula angustifolia* Essential Oil on Anti-*Toxoplasma* Activity. Front. Cell. Infect. Microbiol..

[107] Badreddine B.S., Olfa E., Samir D., Hnia C., Lahbib B.J.M. (2015). Chemical composition of *Rosmarinus* and *Lavandula* essential oils and their insecticidal effects on *Orgyia trigotephras* (Lepidoptera, Lymantriidae). Asian Pac. J. Trop. Med..

[108] Al-Ansari M.M., Andeejani A.M.I., Alnahmi E., AlMalki R.H., Masood A., Vijayaraghavan P., Abdel Rahman A., Choi K.C. (2021). Insecticidal, antimicrobial and antioxidant activities of essential oil from *Lavandula latifolia* L. and its deterrent effects on *Euphoria leucographa*. Ind. Crops Prod..

[109] Radi F.-Z., El Hamzaoui N., Margier A., Auckle T., Oulhaj H., Zair T. (2020). The antibacterial effect of essential oils of *Satureja calamintha* subsp. Nepeta (L.) Briq, *Lavandula multifida* L., and *Mentha pulegium* L., tested against some multiresistant strains that are involved in nosocomial infections. Phytothérapie.

[110] Alabdullatif M., Boujezza I., Mekni M., Taha M., Kumaran D., Yi Q.-L., Landoulsi A., Ramirez-Arcos S. (2017). Enhancing blood donor skin disinfection using natural oils. Transfusion.

[111] Moustafa M.A.M., El-said N.A., Ahmed F.S., Amer A., Awad M. (2025). In Vitro and *Silico* exploration of the insecticidal properties of *Lavandula multifida* L. essential oil and its binary combinations with cyantraniliprole and emamectin benzoate on *Spodoptera frugiperda* (Lepidoptera: Noctuidae). Crop Prot..

[112] El-Bokl M.M. (2016). Toxicity and bioefficacy of selected plant extracts against the mosquito vector *Culex pipiens* L. (Diptera: Culicidae). J. Entomol. Zool. Stud..

[113] Hamad Al-Mijalli S., Elsharkawy E.R., Abdallah E.M., Hamed M., El Omari N., Mahmud S., Bouyahya A. (2022). Determination of volatile compounds of *Mentha piperita* and *Lavandula multifida* and investigation of their antibacterial, antioxidant, and antidiabetic properties. Evid. Based Complement. Alternat. Med..

[114] Mączka W., Duda-Madej A., Grabarczyk M., Wińska K. (2022). Natural Compounds in the Battle against Microorganisms—Linalool. Molecules.

[115] Ramdani C., El Fakhouri K., Sbaghi M., Bouharroud R., Boulamtat R., Aasfar A., Mesfioui A., El Bouhssini M. (2021). Chemical Composition and Insecticidal Potential of Six Essential Oils from Morocco against *Dactylopius opuntiae* (Cockerell) under Field and Laboratory Conditions. Insects.

[116] Henni A., Chahbar M., Tefiel H., Touahri A., Chouhim K.M.A. (2024). Chemical Composition and Efficacy of Essential Oil from *Thymus lancéolatus* Desf. Against *Paenibacillus larvae* and *Ascosphaera apis* in Honeybees (*Apis mellifera*). Braz. J. Anim. Environ. Res..

[117] Khalid S., Keller N.P. (2021). Chemical Signals Driving Bacterial–Fungal Interactions. Environ. Microbiol..

[118] Domingues J., Delgado F., Gonçalves J.C., Zuzarte M., Duarte A.P. (2023). Mediterranean Lavenders from Section *Stoechas*: An Undervalued Source of Secondary Metabolites with Pharmacological Potential. Metabolites.

[119] Zuzarte M., Gonçalves M.J., Cavaleiro C., Dinis A.M., Canhoto J.M., Salgueiro L.R. (2009). Chemical Composition and Antifungal Activity of the Essential Oils of *Lavandula pedunculata* (Miller) Cav. Chem. Biodivers..

[120] Karaca N., Demirci B., Demirci F. (2018). Evaluation of *Lavandula stoechas* L. subsp. *Stoechas* L., *Mentha spicata* L. subsp. *Spicata* L. Essential Oils and Their Main Components against Sinusitis Pathogens. Z. Naturforsch. C.

[121] Zuzarte M., Gonçalves M.J., Cavaleiro C., Canhoto J., Vale-Silva L., Silva M.J., Pinto E., Salgueiro L. (2011). Chemical Composition and Antifungal Activity of the Essential Oils of *Lavandula viridis* L’Hér. J. Med. Microbiol..

[122] Shakeri A., Sahebkar A., Javadi B. (2016). *Melissa officinalis* L.—A review of its traditional uses, phytochemistry and pharmacology. J. Ethnopharmacol..

[123] Deng B., Kong W., Suo H., Shen X., Newton M.A., Burkett W.C., Zhao Z., John C., Sun W., Zhang X. (2023). Oleic Acid Exhibits Anti-Proliferative and Anti-Invasive Activities via the PTEN/AKT/mTOR Pathway in Endometrial Cancer. Cancers.

[124] Kodati B.R., Sampathkumar Y., Godishala V. (2025). Gas Chromatography-Mass Spectrometry Analysis of *Anabaena* Extract: Identification of Bioactive Compounds and Their Therapeutic Potential. J. Food Chem. Nanotechnol..

[125] Fahmy M.A., Farghaly A.A., Hassan E.E., Hassan E.M., Hassan Z.M., Mahmoud K., Omara E.A. (2022). Evaluation of the Anti-Cancer/Anti-Mutagenic Efficiency of *Lavandula officinalis* Essential Oil. Asian Pac. J. Cancer Prev..

[126] Dalilan S., Rezaei-Tavirani M., Nabiuni M., Heidari-Keshel S., Zamanian Azodi M., Zali H. (2013). Aqueous Extract of *Lavender Angustifolia* Inhibits Lymphocytes Proliferation of Hodgkin’s Lymphoma Patients. Iran. J. Cancer Prev..

[127] Zhao Y., Chen R., Wang Y., Qing C., Wang W., Yang Y. (2017). In Vitro and In Vivo efficacy studies of *Lavender angustifolia* essential oil and its active constituents on the proliferation of human prostate cancer. Integr. Cancer Ther..

[128] Ahmed S.B.H., Sghaier R.M., Guesmi F., Kaabi B., Mejri M., Attia H., Laouini D., Smaali I. (2011). Evaluation of Antileishmanial, Cytotoxic and Antioxidant Activities of Essential Oils Extracted from Plants Issued from the Leishmaniasis-Endemic Region of Sned (Tunisia). Nat. Prod. Res..

[129] Rathod N.B., Kulawik P., Ozogul F., Regenstein J.M., Ozogul Y. (2021). Biological activity of plant-based carvacrol and thymol and their impact on human health and food quality. Trends Food Sci. Technol..

[130] Bertelli A., Biagi M., Corsini M., Baini G., Cappellucci G., Miraldi E. (2021). Polyphenols: From Theory to Practice. Foods.

[131] Dias M.C., Pinto D.C., Silva A.M. (2021). Plant Flavonoids: Chemical Characteristics and Biological Activity. Molecules.

[132] Sales-Campos H., de Souza P.R., Peghini B.C., da Silva J.S., Cardoso C.R. (2013). An Overview of the Modulatory Effects of Oleic Acid in Health and Disease. Mini-Rev. Med. Chem..

[133] Aryal P., Syed I., Lee J., Patel R., Nelson A.T., Siegel D., Saghatelian A., Kahn B.B. (2021). Distinct Biological Activities of Isomers from Several Families of Branched Fatty Acid Esters of Hydroxy Fatty Acids (FAHFAs). J. Lipid Res..

[134] He Y., Wang Y., Yang K., Jiao J., Zhan H., Yang Y., Lv D., Li W., Ding W. (2022). Maslinic Acid: A New Compound for the Treatment of Multiple Organ Diseases. Molecules.

[135] Wimmer Z. (2025). Selected Pentacyclic Triterpenoids and Their Derivatives as Biologically Active Compounds. Molecules.

[136] de Sousa D.P., Damasceno R.O.S., Amorati R., Elshabrawy H.A., de Castro R.D., Bezerra D.P., Nunes V.R.V., Gomes R.C., Lima T.C. (2023). Essential Oils: Chemistry and Pharmacological Activities. Biomolecules.

